# Discrete regulatory modules instruct hematopoietic lineage commitment and differentiation

**DOI:** 10.1038/s41467-021-27159-x

**Published:** 2021-11-23

**Authors:** Grigorios Georgolopoulos, Nikoletta Psatha, Mineo Iwata, Andrew Nishida, Tannishtha Som, Minas Yiangou, John A. Stamatoyannopoulos, Jeff Vierstra

**Affiliations:** 1 Altius Institute for Biomedical Sciences, Seattle, WA USA; 2 Department of Genetics, Development & Molecular Biology, School of Biology, Aristotle University of Thessaloniki, Thessaloniki, Greece; 3 Department of Genome Sciences, University of Washington, Seattle, WA USA; 4 Division of Oncology, Department of Medicine, University of Washington, Seattle, WA USA

**Keywords:** Haematopoiesis, Epigenomics, Gene regulation, Stem-cell differentiation, Haematopoietic stem cells

## Abstract

Lineage commitment and differentiation is driven by the concerted action of master transcriptional regulators at their target chromatin sites. Multiple efforts have characterized the key transcription factors (TFs) that determine the various hematopoietic lineages. However, the temporal interactions between individual TFs and their chromatin targets during differentiation and how these interactions dictate lineage commitment remains poorly understood. Here we perform dense, daily, temporal profiling of chromatin accessibility (DNase I-seq) and gene expression changes (total RNA-seq) along ex vivo human erythropoiesis to comprehensively define developmentally regulated DNase I hypersensitive sites (DHSs) and transcripts. We link both distal DHSs to their target gene promoters and individual TFs to their target DHSs, revealing that the regulatory landscape is organized in distinct sequential regulatory modules that regulate lineage restriction and maturation. Finally, direct comparison of transcriptional dynamics (bulk and single-cell) and lineage potential between erythropoiesis and megakaryopoiesis uncovers differential fate commitment dynamics between the two lineages as they exit the stem and progenitor stage. Collectively, these data provide insights into the temporally regulated synergy of the *cis*- and the *trans*-regulatory components underlying hematopoietic lineage commitment and differentiation.

## Abstract (web summary)

Lineage differentiation and commitment is driven by transcription regulators and chromatin changes. Here the authors report daily profiling of chromatin accessibility and transcriptome changes during human erythropoiesis, relating these changes to lineage potential between erythropoiesis and megakaryopoieis.

## Introduction

The temporal activation of stage-specific regulatory DNA instructs lineage specific gene expression programs that underpin cellular fate and potential. The establishment and maintenance of regulatory DNA is mediated by the combinatorial engagement of sequence-specific transcription factors (TFs) that bind in the place of a canonical nucleosome. Over the course of cellular differentiation programmed shifts in the global transcription factor milieu drive extensive re-organization of chromatin^1,2^, where silencing of regulatory DNA associated with alternate lineage and the de novo activation of lineage-restricted elements result in the narrowing of the epigenetic and functional landscape^3^. However, it is unclear how and when regulatory DNA is dynamically activated and silenced during cell state transitions to establish lineage restricted gene expression programs and how these epigenetic changes relate to developmental potential.

Hematopoiesis is a prototypical system to study how genetically and epigenetically encoded programs are established during cellular differentiation^4–6^. Conventionally, hematopoiesis is depicted as a discrete hierarchical process where a multipotent hematopoietic stem and progenitor cell (HSPC) traverses a sequence of bifurcating decisions, mediated by the expression of lineage-specific TFs, with each decision resulting in an increasingly restricted fate potential. Historically, the characterization of the gene regulatory programs involved in the transition from HSPCs to terminal fates has relied on the identification of differential transcriptional programs from isolated discrete populations using defined cell surface markers^7–10^. While this approach has led to the identification of master regulatory transcription factors^10,11^ that define many of the major hematopoietic cell lineages and has enabled a systematic mapping of their steady-state regulatory landscapes^9,12^, interrogation of discretely defined populations cannot elucidate the dynamic regulatory events that mark cell-state transitions.

Recently, single-cell chromatin and transcriptional profiling assays have attempted to resolve the spatio-temporal *cis*- and *trans*- dynamics in different stages of hematopoiesis^13–16^. These studies have largely relied on the analysis of either bulk or immunophenotypically isolated populations of steady-state peripheral blood or bone marrow derived cells, whereby hierarchical relationships and developmental trajectories between cell states are predicted computationally. While such experimental approaches have aided in defining major subpopulations of hematopoietic cells and their respective epigenetic and transcriptional landscapes, definition of developmental trajectories within individual lineages from population snapshots is challenging due to the limited sensitivity and the resulting technical and analytical artifacts associated with single-cell genomic assays^17,18^. Additionally, because developmental trajectories are predicted in silico, direct association of functional changes (i.e., lineage potential) to intermediate cellular states is not possible^19^.

In order to investigate the dynamics of regulatory and functional events during differentiation, we use human erythropoiesis as a proxy for hematopoietic development. The transition from HSPCs to terminally differentiated enucleated red blood cells involves a series of morphologically, functionally, and phenotypically distinguishable states. Multiple efforts relying on the isolation of these states have exhaustively characterized key transcriptional regulators^20,21^ and chromatin elements implicated in erythropoiesis^9,22^. However, a general understanding of the temporal interplay between individual *cis*- and *trans*- elements and how these establish stage-specific transcriptional programs and lineage commitment during hematopoiesis remains rudimentary. Furthermore, because erythrocytes share their developmental origins with other myeloid lineages (granulocytic/monocytic and megakaryocytic), erythropoiesis represents an ideal system to study how lineage choice is genetically and epigenetically encoded.

Here, we capitalize on the ex vivo human differentiation scheme where dense unbiased sampling of the populations captures the dynamics of chromatin accessibility and gene expression during differentiation with a completely defined developmental trajectory. DNase I-seq and gene expression profiling (bulk and single cell) time-course during erythropoiesis coupled with lineage potential assays and morphological characterization, enabled the assignment of distal elements (alone or in combination) to target genes and individual TFs to their target DHSs which collectively comprise discrete regulatory modules associated with lineage potential. Comparing the activity patterns of the TF regulatory modules in the erythroid lineage to the closely related megakaryocytic lineage, provides insights into how these modules instruct lineage commitment. Collectively, our findings provide key insights into the organization of the functional epigenetic landscape during hematopoietic differentiation and its relation to lineage-potential.

## Results

### A temporal atlas of chromatin and transcriptional dynamics

**Fig. 1:**
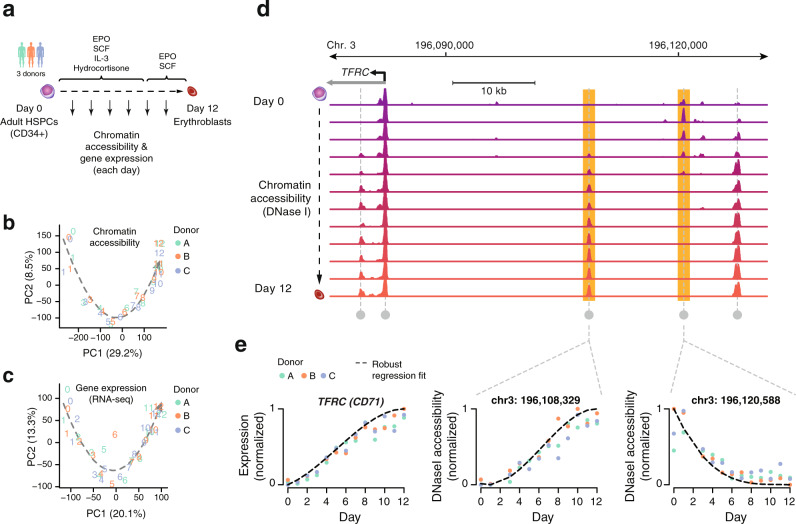
Comprehensive identification of regulatory landscape developmental dynamics. **a** Dense DNase I-seq and RNA-seq time course with daily sampling during the 12-day ex vivo erythroid differentiation induced from CD34^+^ HSPCs. **b** PCA analysis using all detected DHSs (79,085 Hotspots 5% FDR) across all samples (12 time points, 3 donors). The arrow denotes the differentiation trajectory from day 0 to day 12. **c** PCA analysis using all 24,849 detected genes across all samples (13 time points, 3 donors). The arrow denotes the differentiation trajectory from day 0 to day 12. **d** Chromatin accessibility tracks for each day of differentiation with DNase Hypersensitive Sites (DHSs) harbored around the *TFRC* locus. **e** Identification of significantly changing DHS and genes with robust linear regression analysis. Scatterplots show *TFRC* expression and DNase I density for two upstream DHS. Dots represent normalized values for each of the 3 donors. Dashed line represents the fitted regression spline.

Human erythropoiesis was induced ex vivo for 12 days using an established differentiation protocol^23^ that faithfully recapitulates the major features of in vivo erythropoiesis. Starting from human adult-derived mobilized peripheral blood CD34^+^-enriched HSPCs from 3 healthy donors we cultured the cells in defined media for 12 days (Fig. 1a and Methods). Characteristic features of developing erythroblast cells were confirmed by immunophenotyping using canonical cell-surface markers of early (CD117, C-Kit) and late (CD235a, Glycophorin A) erythropoiesis as well as morphologically by hematoxylin-eosin staining of cell smears (Supplementary Fig. 1).

To densely map both chromatin accessibility and transcriptional dynamics during the transition from HSPCs to committed erythroblasts, we subsampled a single continuous culture each day (12 days) and performed DNase I-seq analysis and total RNA-seq (Fig. 1a). Biological replicates from CD34^+^ HSPCs from 3 donors were highly reproducible for both chromatin accessibility and gene expression profiles where the majority of the observed variability was accounted for by developmental trajectory (i.e., sampling days) (Fig. 1b, c and Supplementary Fig. 2a), as biological replicates were highly correlated (Supplementary Fig. 2b, c). For many individual DHSs and genes, we observed quantitative changes in chromatin accessibility and expression over the course of differentiation highlighted by quantitative trajectories of opening or closing (Fig. 1d, e). Notably, accessibility changes were mostly confined to compact regions of the genome (~200 bp average DHS width). In many cases, we observed both opening and closing events within close proximity (Fig. 1d), indicating focal regulation^24^ of chromatin structure in contrast to previous reports that chromatin changes during differentiation occur over large domains^1,25^.

To systematically identify developmentally responsive *cis*-elements, we leveraged the observed continuity of DHS signal over adjacent days (Fig. 1d) and modeled DNase I cleavage density against differentiation time-points (Methods). We determined significance by comparing our full model to a reduced model (intercept-only; not accounting for developmental time) and performing a likelihood ratio test (Methods). Of the total 79,085 DHSs accessible in 2 or more samples/replicates, we conservatively identified 11,805 (14.9%) significantly changing DHSs (adjusted *p* < 10^−5^ and fold-change >2), nearly evenly grouped between activated and silenced (45% and 55%, respectively) (Supplementary Data 1). A similar analytical approach applied to the RNA expression data identified 5769 developmentally regulated genes (adjusted *p* < 10^−5^ and fold-change >2), of which 62% upregulated and 38% downregulated over the course of differentiation (Supplementary Data 2). Collectively, these data define a high-resolution and quantitative map of chromatin and gene expression dynamics during erythroid differentiation.

### Stage-specific *cis*- and *trans*- regulatory compartments

**Fig. 2:**
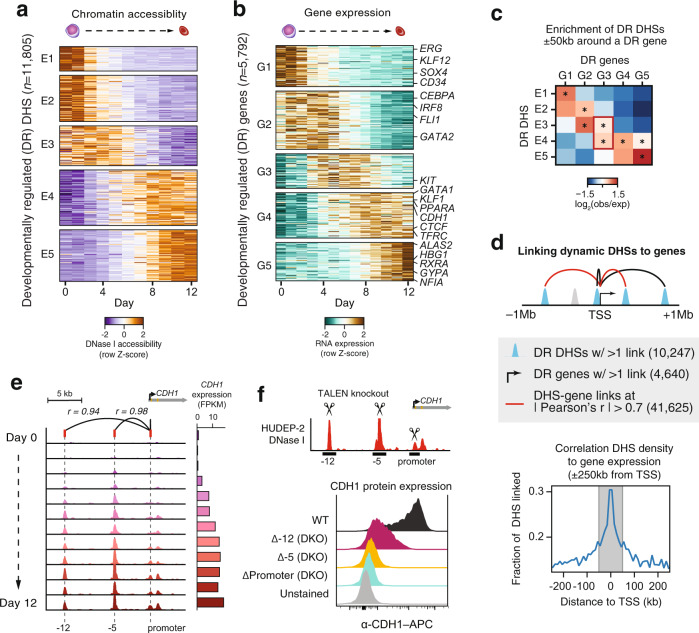
Temporal compartmentalization of the *cis-* and *trans*- regulatory landscape during erythropoiesis. **a** *K*-means clustering of 11,805 changing DHS resulted in 5 clusters (E1-E5) with sequential activity profiles for each cluster. Values are *z*-score of per day average normalized DHS counts from 3 donors. **b** *K*-means clustering of 5792 developmentally regulated genes resulted in 5 clusters (G1–G5). Values are z-score of per day average normalized FPKM from 3 donors. **c** A matrix showing the enrichment score (log2-ratio observed over expected) for any given DHS cluster, around each developmentally regulated DHS ( ± 50 kb from TSS). Highlighted in red is cluster G3 which is enriched for both late downregulated DHS of cluster E3 and early upregulated from cluster E4. Asterisks denote *X*^2^ test *p*-value < 0.05. **d** Correlation density plot between developmental genes and developmental DHS ± 250 kb around the gene promoter. Gray shaded area highlights enrichment of correlations within ±50 kb around the gene promoter. **e** DNase I accessibility track of the *CDH1* locus during erythroid differentiation, highlighting the accessibility of 3 nearby DHS correlated to *CDH1* expression. **f** DNase I accessibility track of the *CDH1* locus in HUDEP-2 cells depicting the genetic knockout of the *CDH1* promoter and two upstream DHS (-12, and -5) (above) along with the resulted ablation in CDH1 protein expression as assessed by flow cytometry (below).

PCA indicated that days 5–6 were associated with a critical developmental inflection point during ex vivo differentiation (Fig. 1b, c). We therefore sought to characterize the relationship between temporal chromatin and gene expression dynamics with regards to the observed immunophenotypic and morphological changes present in the population of differentiating cells. We performed unsupervised clustering (*K*-means; *k* = 5) on dynamically changing DHSs and developmentally responsive transcripts (Fig. 2a, b). This analysis revealed a stark partitioning of activated and silenced genes and DHSs into non-overlapping sets that closely paralleled canonical developmental features of erythropoiesis. Particularly, DHSs rapidly silenced within the first days of differentiation (clusters E1 and E2) were found to preferentially harbor binding sequences utilized by the known HSPC regulators such as (HOXA9^26^, RUNX^27^, and ERG^28^) (Supplementary Fig. 3). Similarly, immediately downregulated transcripts upon induction of differentiation (cluster G1) include these transcription factors as well as structural genes characteristic of CD34^+^ HSPCs (Fig. 2b). Consistent with PCA (Fig. 1b, c), a rapid and marked turnover of chromatin and gene expression landscape is observed between days 5–7 where an early erythroid signature appears in both activated DHSs and gene expression, marked by the upregulation of *GATA1*, *KLF1*, *PPARA*, and *TFRC* (cluster G4). Markers of mature erythropoiesis emerge later in the differentiation (after day 8; cluster G5) with the upregulation of hemoglobins, glycophorin A (*GYPA*) and *ALAS2* (Fig. 2b). Beyond the temporal partitioning of developmentally regulated DHS and transcripts we observed topological segregation of co-regulated elements. Mapping changing DHS and genes to TADs called from CD34^+^ HSPCs^29^ (Supplementary Data 3) and day 11 ex vivo differentiated erythroid progenitors^30^ (Supplementary Data 4) Hi-C data revealed enrichment of individual TADs for stage-specific elements (Supplementary Fig. 3). Additionally, this partitioning appears more contrasted in late erythroid TADs compared to CD34^+^ , suggesting the establishment of a defined erythroid regulatory landscape.

In addition to canonical activation and downregulation patterns observed, we found a subset of genes exhibiting reproducible transient upregulation (clusters G2 and G3) occurring prior to establishment of the erythroid signature (Fig. 2b). Transiently upregulated genes are found enriched in transcripts representing myeloid lineages including several myeloid markers (e.g., *MPO*, *KIT*) as well as the myeloid-specific transcription factor CEBPA. Compatible with gene expression, late closing DHSs in cluster E2 and E3 were enriched in CEBPA recognition sequences (Supplementary Fig. 3, Supplementary Data 5). Moreover, the majority (~80%) of DHSs in cluster E2 and E3 were found overlapping with DHSs active in other myeloid cell types (macrophages and monocytes) (Supplementary Data 5), denoting a transient emergence of myeloid-related regulatory program prior to erythroid commitment.

Taken together these data describe the sequence of developmentally related changes in both the *cis*- and *trans*- environment as the regulatory landscape of the erythroid development traverses from a lineage-permissive program to a defined erythroid-specific signature. Expectedly, activated DHSs (clusters E4-E5) were found to preferentially harbor red blood cell-related GWAS variants (1.36-fold enrichment over all detected DHSs), highlighting their functional role in regulating erythropoiesis (Supplementary Fig. 6).

### Connecting individual DHSs to genes

The overall dynamics of chromatin accessibility for individual DHSs closely mirrored that of the expression of nearby genes. To formulate this, we performed an enrichment test to investigate the DHS landscape around a gene promoter. Interestingly, we found that developmentally regulated genes are enriched for DHSs with a similar developmental profile (Fig. 2c). For example, early closing genes (cluster G1) are significantly enriched for cluster E1 DHSs. Noteworthy, transient genes of cluster G3 are harboring DHSs belonging to both late closing DHS cluster E3 and early activated erythroid DHS cluster E4, suggesting that the transient nature of these genes is a result of the combinatorial activity of a closing and an opening chromatin landscape.

Because of fine-resolution afforded by our dense sampling approach, we sought to quantify the extent of genome-wide coactivation patterns that could potentially comprise physical regulatory links between DHSs and their target genes. To this aim we correlated the temporal expression patterns of a gene to nearby (±1 Mb from TSS) developmentally regulated DHSs, given that the majority of transcriptional enhancers are located within this range from the target promoter^31^. This analysis identified 41,625 connections (absolute Pearson correlation coefficient *r* > 0.7), with the vast majority of gene-DHS links occurring within 50 kilobases of the transcription start site (Fig. 2d). Overall, we connected 80.4% (4640) of the developmentally regulated genes with ≥1 DHS and 86.8% (10,247) of changing DHSs were linked to ≥1 developmentally regulated gene (Supplementary Data 6). While on average 93.6 DHSs reside within ±1 Mb of a given gene, only 9 DHSs (±8 SD) were found to be linked with a changing gene. This allowed us to identify *cis*-regulatory inputs at much higher resolution than typically afforded by standard chromatin conformation-based methods^32,33^. For example, using previously published Hi-C data derived from ex vivo cultured erythroid progenitors we were able to predict chromatin loops only down to 70 kb (Supplementary Fig. 7, Supplementary Data 7). In order to functionally validate these associations we performed genetic perturbation of gene-DHS links.

We focused on the *cis*-elements predicted to regulate the expression of *CDH1*. CDH1 is a cell surface marker with expression restricted to erythropoiesis among the hematopoietic populations^34^ and known to be implicated in erythroid development and maturation^35,36^. Specifically, we genetically disrupted two DHSs (HS1 and HS2) highly correlated with *CDH1* expression (*r* = 0.939 and 0.976, respectively), situated upstream (5 kb and 12 kb, respectively) of the promoter of *CDH1* using TALE-nucleases^37,38^ (Fig. 2e and Supplementary Fig. 8). Homozygous deletion of these DHSs as well as the promoter in the human-derived erythroid progenitor cell line HUDEP-2 where these DHSs are also active, resulted in complete ablation of the *CDH1* expression as determined by flow-cytometry (Fig. 2f). These results suggest that both elements predicted by the correlation analysis as transcriptional regulators of *CDH1*, indeed drive the expression of the gene and their deletion confers effects similar to the deletion of the gene promoter.

Overall, these findings suggest that the majority of changes in transcription during development are regulated by a limited number of *cis*-regulatory inputs, situated within close proximity to the genes they regulate.

### Sequential regulatory modules encode differentiation stages

**Fig. 3:**
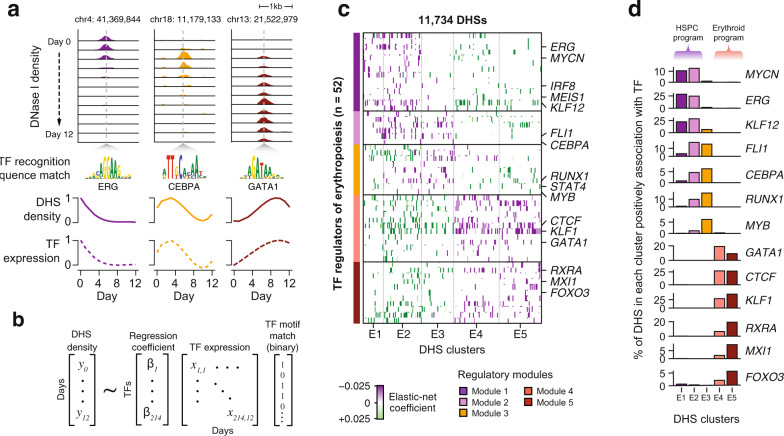
Systematic modeling of *cis*- and *trans*- element temporal interactions reveals discrete regulatory modules spanning erythropoiesis. **a** Developmental responses of DHS accessibility and transcription factor expression levels were found to be correlated across the genome. **b** The density of a given developmental DHS is modeled after the TF binding motif composition and the expression of the binding TFs using elastic-net regression. The model returns a coefficient for each pair of DHS and binding TF which denotes how strongly (positively or negatively) the TF expression is associated with the accessibility of the particular DHS. **c** Hierarchical clustering of 52 highly connected TFs based on the cosine distances of the regression coefficient from 11,734 DHS reveals 5 clusters of developmentally regulated TFs. Transcription factors along with their positively associated DHS comprise a regulatory module (modules 1–5). **d** The fraction of DHS per cluster positively associated with a TF identifies the major drivers of chromatin accessibility during erythropoiesis.

Clustering of dynamically changing DHSs revealed that chromatin activated at different stages of hematopoiesis display differential enrichment for transcription factor recognition sequences, indicating stage-specific regulation of *cis*-elements. This, however, does not resolve the temporal interactions between individual DHSs and individual *trans*-regulators and how this relationship shapes the developmental response of a DHS. Given the observed global correlated changes between the transcription factor expression levels and the accessibility of the DHSs containing their cognate recognition sequences (Fig. 3a) we sought to quantify the contribution of individual TFs to the dynamic changes in DNase I density at individual regulatory *cis*-elements. We capitalized on our dense sampling approach and applied a regression strategy where the activity of an individual regulatory element (i.e., DNase I cleavage density) is modeled as a function of the gene expression profiles of developmentally regulated TFs with a compatible recognition sequence harbored within each DHS (Fig. 3b and Methods). We controlled for weak and ambiguous association of TFs recognizing degenerate motifs using elastic-net regularization (Methods). We applied this approach to all of the 11,805 dynamically changing DHSs, identifying 11,734 (>99% of changing DHSs) with at least one explanatory TF regulator (Methods) and 88 developmentally regulated TFs associated with at least one DHS, where the regression coefficients broadly correspond to the strength of association of a TF with an individual DHS (Supplementary Fig. 9). Overall, 5 TFs on average were positively associated with each DHS, suggesting that a small subset of TFs regulate the developmental activity of individual *cis*-elements. We then evaluated whether the regression results hold any predictive capacity against the frequency of motifs for the same TFs. Using a naïve Bayes classification model (Methods) we tried to predict the cluster each DHS belongs to by supplying either the occurrences of individual TF motifs or the elastic-net regression coefficients for each TF. We found that elastic-net regression coefficients provide a 1.77-fold accuracy over TF motif counts (62% vs. 35% accuracy rate) in predicting the DHS cluster (Supplementary Fig. 10). This suggests that the developmental response of a DHS is shaped by co-regulated transcription factors that occupy the DHS rather than the absolute frequency of binding TFs as determined by the TF recognition sequences harbored in a DHS.

We next asked to what extent the activity of DHSs with similar temporal accessibility patterns are regulated by a coherent set of TF regulators. We selected 52 TFs positively associated with at least 200 DHSs and performed unsupervised hierarchical clustering based on their regression coefficients computed for each DHS (Fig. 3c and Methods). This analysis resolved the temporal associations between transcription factors and their target DHS into a sequence of five discrete and largely non-overlapping regulatory modules, reflective of developmental stages of erythropoiesis (Fig. 3d). Module 1 consists of known HSPC transcriptional regulators (e.g., ERG^28^, MEIS1^39^, MYCN^40^) which are positively associated with early closing DHSs in clusters E1 and E2. In modules 2 and 3, transcription factors associated with commitment of hematopoietic progenitors to the different myeloid lineages (e.g., CEBPA^41^, MYB^42^, FLI1^43^, RUNX1^44^) interact with DHSs in clusters E2 and E3. Modules 4 and 5 define the erythroid-specific regulatory landscape as known erythroid regulators (e.g., GATA1^45^, KLF1^20^, RXRA^46^, and FOXO3^47^) positively interact with activated DHSs in clusters E4 and E5.

Plotting the fraction of DHSs in each cluster positively associated with each TF (Fig. 3d) highlights the major drivers of chromatin accessibility in each developmental stage. Particularly, ERG appears as a major regulator of the HPSC stage as it is positively associated with ~25% of DHSs in clusters E1 and E2. Although ERG has been long implicated in HSPC regulation, it is only recently its role as a critical regulator of HSPC survival has been appreciated^48^. Interestingly, KLF12 also appears to share a significant proportion of the early chromatin landscape, although its role in HSPC regulation is not fully elucidated. Overexpression of the critical HSC regulator Evi-1 in mice, resulted in maintenance of the quiescent phenotype of murine HSCs along with the more than 12-fold increase in *Klf12* expression^49^. In another experiment, sustained expression of *Hlf* in mice also resulted in enrichment of *Klf12* in more primitive hematopoietic compartments^50^, thus implicating KLF12 in the HSPC regulation. Apart from the canonical erythroid transcription factors, we identified MXI1 among the top regulators of the erythroid chromatin landscape. Knockdown of *Mxi1* in mice, blocks chromatin condensation and impairs enucleation of mouse erythroblasts, highlighting the role of MXI1 in erythroid maturation^51^. Additionally, we find CTCF to be positively associated with a large portion of the erythroid-specific chromatin (~25% of DHSs in clusters E4 and E5), while we find strong enrichment for DHS harboring CTCF motifs in predicted chromatin loops from Hi-C data generated from ex vivo cultured human erythroid progenitors (Supplementary Fig. 11). These findings are consistent with the evidence highlighting the role of CTCF in establishing the erythroid-specific chromatin landscape^52,53^.

Taken together, these findings illustrate the dynamic interaction of the *cis*- and the *trans*- regulatory landscape during erythropoiesis and their organization into well-defined and discrete regulatory modules of associated DHSs with their cognate transcription factors, reflecting distinct stages of erythroid development.

### Abrupt lineage restriction events mark erythropoiesis

**Fig. 4:**
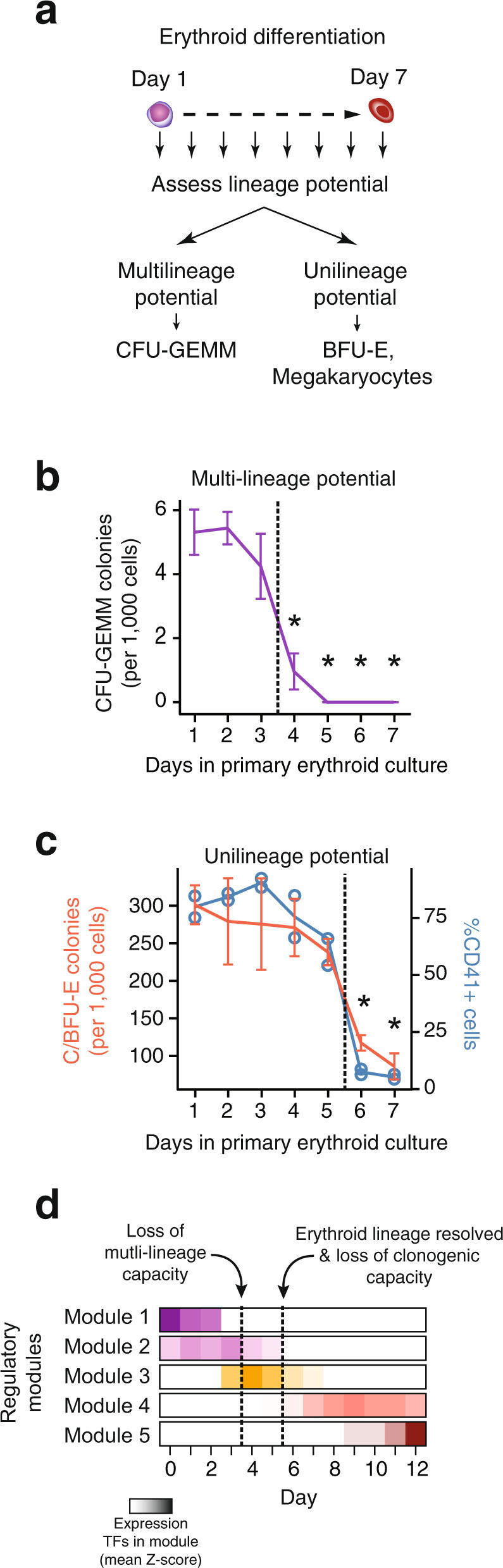
Lineage restriction events during erythropoiesis reflect the sequence of regulatory programs. **a** Schematic diagram of lineage potential assays during the first 7 days of ex vivo erythropoiesis. Cells were sampled daily and transferred to lineage-permissive media. Multilineage capacity was determined as frequency of CFU-GEMM progenitors. Erythroid potential as frequency of BFU-Es and megakaryocytic potential as frequency of CD41^+^ cells. **b** Frequency of multipotent CFU-GEMM in methylcellulose assay from cells sampled over the course of erythroid differentiation. **c** Frequency of unipotent erythroid progenitor colonies (BFU-E) in methylcellulose assay (red line) and frequency of CD41^+^ cells after transplantation in secondary megakaryocytic media (blue line). **d** Changes in lineage potential coincide with the transitions between the regulatory modules identified earlier. Transition from modules 1 and 2 to module 3 reflects the loss of multipotency occurring between days 3 and 4, while transition from module 3 to erythroid modules 4 and 5 coincides with the depletion of unipotent progenitors and entry to erythroid maturation (days 5 to 6). Data represent mean values. Error bars denote ±1 SE of the mean from *n* = 4 experiments. Asterisks denote statistically significant difference in CFU-GEMM and BFU-E counts from day 1 (two-sided Student’s *t* test *p*-value <0.05). CFU-GEMM Colony Forming Unit - Granulocytic, Erythroid, Macrophage, Megakaryocyte. BFU-E Burst Forming Unit-Erythroid.

The organization of chromatin and transcription factors into defined regulatory modules corresponding to distinct stages of erythropoiesis indicates a functional relationship between lineage potential and module activity. To gain insight into whether these modules underpin lineage decision events we determined the lineage potential of the erythroid cultures by daily sampling a population of cells and assaying their multipotent and unipotent capacity for different myeloid lineages (Fig. 4a and Supplementary Fig. 12a). Total number of colonies declined with the progress of differentiation resulting in an abrupt depletion of total progenitors on day 6 of differentiation (Supplementary Fig. 12b). After 4 days of exposure to erythroid media, the most primitive and multipotent colonies (CFU-GEMM; granulocytic, erythroid, monocytic, megakaryocytic) were no longer detected (Fig. 4b). Day 6 marked a second event of restriction of the fate potential as all unilineage colonies were no longer detected in the cultures. Specifically, frequency of erythroid progenitors (BFU-E) rapidly declined from day 5 to day 6 (Fig. 4c). Similarly, granulocytic/monocytic progenitors (CFU-GM) were depleted by day 6 of erythroid differentiation (Supplementary Fig. 12c). Notably, none of the changes in clonogenic capacity were associated with any changes in the growth rate of the parental erythroid cultures, which remained constant throughout the differentiation (Supplementary Fig. 12d), suggestive of an independent mechanism regulating this shift in progenitor population.

In addition to the above lineages, we specifically tested whether cells during erythroid development maintain the capacity to differentiate towards the megakaryocytes by transferring cells, on a daily basis, from the primary erythroid culture to megakaryopoiesis-inducing suspension cultures and tested for their ability to give rise to CD41^+^ megakaryocytic populations (Supplementary Fig. 13a and Methods). Consistent with the overall lineage restriction observed during colony-forming assays, erythroid cultures completely lose megakaryocytic potential on day 6 of the differentiation (Fig. 4c and Supplementary Fig. 13b).

The rapid changes observed in clonogenic capacity correspond to the transitions between regulatory modules (Fig. 4d). Early depletion of primitive multipotent CFU-GEMM progenitors is concomitant with the transition from the HSPC-related modules (modules 1 and 2), while the decline of unipotent progenitors of all detectable myeloid lineages (granulocytic/monocytic, erythroid, megakaryocytic) coincides with the transition from a program with a broader myeloid signature to erythroid specific *cis*- and *trans*- landscape. Furthermore, because these rapid lineage restriction events are not associated with other abrupt changes in morphology or cell growth (Supplementary Fig. 13d), these data suggest that the mechanism responsible for the exit from the progenitor stage is decoupled from maturation progress.

### A shared transcriptional program facilitates HSPC exit

The silencing of the HPSC regulatory modules prior to lineage commitment suggested that exit from the progenitor state is necessary for erythroid commitment to proceed. We therefore asked whether this represents a canonical feature of hematopoietic development to any lineage. To investigate this, we focused on megakaryocytic differentiation, a process that shares both close common developmental origins^54^ and key TF regulators with erythropoiesis^55^.

**Fig. 5:**
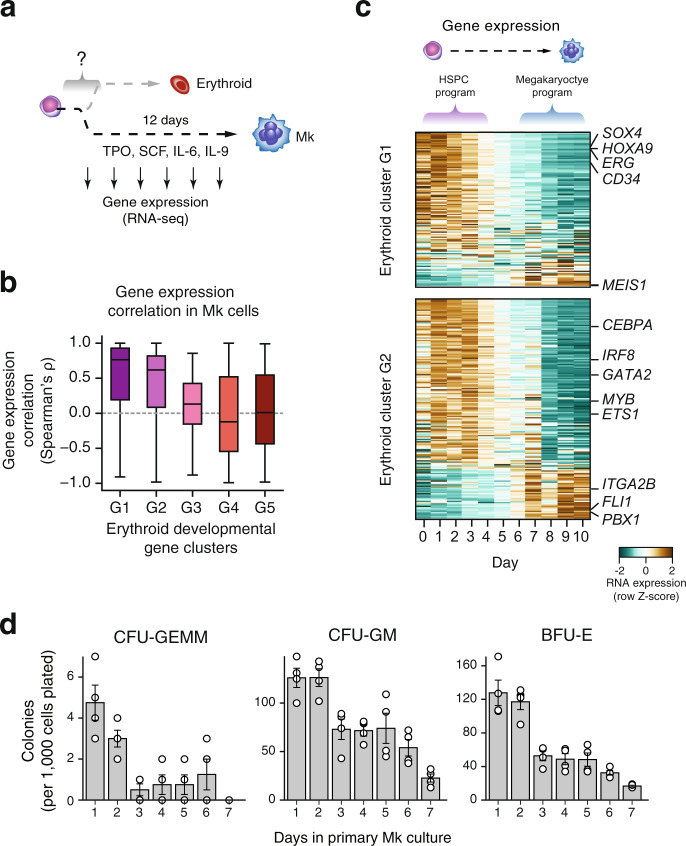
A shared transcriptional program drives the exit from HSPC state early in erythropoiesis and megakaryopoiesis. **a** Dense RNA-seq time course during ex vivo megakaryopoiesis induced from CD34^+^ HSPCs. **b** Boxplot depicting the distribution of pairwise Pearson’s *r* correlation values of gene expression profiles between erythropoiesis and megakaryopoiesis across the erythroid gene clusters G1–G5. Boxplot lower and upper bounds correspond to 25^th^ and 75^th^ percentile, respectively. Centre corresponds to median. Boxplot whiskers correspond to 1.5 x inter-quartile range excluding outliers. (G1 *n* = 1086, G2 *n* = 1544, G3 *n* = 919, G4 *n* = 1203, G5 *n* = 1017 genes tested). **c** Expression profiles during megakaryocytic development, ordered by their correlation score to their erythroid counterparts from erythroid clusters G1 and G2. **d** Changes in clonogenic capacity observed during megakaryocytic differentiation as determined by frequency of CFU-GEMM, CFU-GM, and BFU-E progenitors. Data are presented as mean values ±1 SEM of *n* = 4 experiments. CFU-GEMM Colony Forming Unit - Granulocytic, Erythroid, Macrophage, Megakaryocyte. CFU-GM Colony Forming Unit – Granulocyte-Macrophage. BFU-E Burst Forming Unit-Erythroid.

We induced ex vivo megakaryocytic differentiation and performed dense sampling of gene expression during development (Fig. 5a and Methods). Developmentally regulated genes during megakaryopoiesis exhibit largely bipartite profiles similar to those observed during erythropoiesis (Supplementary Fig. 14, Supplementary Data 8). To determine whether the transcriptional changes associated with exit from HSPC state during erythropoiesis are shared with megakaryopoiesis we examined the expression profiles of erythroid developmentally regulated genes during megakaryocytic differentiation. We observed highly correlated global expression profiles for early silenced transcripts (erythroid clusters G1 and G2) between the two lineages (median Spearman’s *⍴* = 0.76 and 0.62, respectively) (Fig. 5b), with the exception of key regulators and canonical markers of megakaryopoiesis (*MEIS1*, *FLI1, PBX1, ITGA2B*, etc.) (Fig. 5c). In contrast, correlation for erythroid clusters G3–G5 was low (median Spearman’s *⍴* ≤ 0.13).

Similar to erythropoiesis, we found that early downregulation of HSPC-related gene signature is associated with abrupt restriction of alternate lineage potential during megakaryopoiesis. Specifically, we found that cells sampled beyond day 3 of differentiation exhibit a reduction in both multipotent and unipotent progenitors of the erythroid and granulocytic/monocytic lineage (Fig. 5c and Supplementary Fig. 15). This observation is in line with the fact that megakaryopoiesis does not exhibit transient activation of myeloid gene program as exit from HSPC is rapidly succeeded by a megakaryocyte-specific gene signature, compatible with the recently revised hematopoietic tree according to which megakaryocytes directly emerge from the primitive HSPC compartments bypassing the common myeloid progenitor^56–58^.

Conclusively, these results indicate the existence of a shared mechanism between erythropoiesis and megakaryopoiesis driven by a common set of TFs which mediates the exit from HSPC state signaling differential lineage potential response for erythropoiesis and megakaryopoiesis.

### Myeloid promiscuity precedes erythroid commitment

**Fig. 6:**
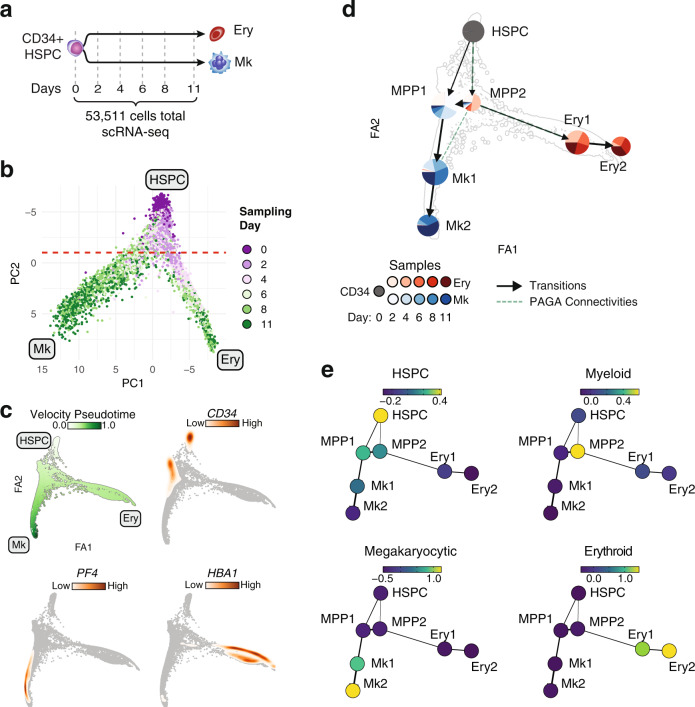
Single-cell gene expression dynamics demonstrate distinct cell states during erythroid and megakaryocytic differentiation. **a** Schematic diagram of the single-cell gene expression timecourse experiment along ex vivo erythropoiesis and megakaryopoiesis. **b** PCA using the top 2000 variable genes. Cells are colored by sampling day. Terminal erythroid (Ery) and megakaryocytic (Mk) states, as well as initial HSPC state are annotated. Red line denotes where 90% of the cells sampled prior to day 4 are located (above line). **c** RNA velocity-based pseudotime or gene expression density on Force-Atlas graph of all cells based on RNA velocity estimates of top 2000 variable genes. **d** Cell populations from collapsed Leiden clusters on FA graph. Sample composition of each population is represented as a pie chart. Arrows denote RNA velocity derived transitions between populations. Dashed lines represent PAGA connectivities. **e** Average expression intensity of gene sets representative of HSPC, Myeloid, Erythroid, and Megakaryocytic states on identified cell populations.

Genomic and functional findings on population-level during ex vivo erythropoiesis suggest that erythroid development transitions through a state with permissive alternate lineage potential prior to erythroid commitment whereas megakaryocytes appear to rapidly commit after exit from HSPC. As lineage decision events resolve in individual progenitors, we sought to chart fate commitment kinetics and the differentiation trajectories of erythropoiesis and megakaryopoiesis by jointly analyzing transcriptional dynamics in single cells along the two lineages. To this end, we analyzed transcriptional changes from more than 50,000 single cells sampled from frequent intervals along both the ex vivo erythroid and megakaryocytic differentiation (Fig. 6a). Overall, we found that single-cell gene expression profiles to be highly concordant with total RNAseq data as aggregated gene expression from single-cell RNA-seq correlated very well with RNA-seq performed in bulk cells (Supplementary Fig. 16).

Principal component analysis (PCA) using the top 2000 variable genes readily resolved the two primary axes of differentiation. Trajectories from HSPC to terminally committed lineages are resolved along PC2 while PC1 distinguishes the erythroid and megakaryocytic terminal fates (Fig. 6b). Furthermore, PCA highlights the lineage commitment timepoint as cells sampled on day 4 and thereon, from either culture, already exhibit distinct topologies on the PCA projection. In order to infer rate of transcription and derive the direction of differentiation, we capitalized on splicing kinetics (RNA velocity) to derive latent pseudotime (Fig. 6c). Overall, pseudotime correlated well with actual sampling time (Pearson’s *r* = 0.725).

Cells were clustered using Leiden community detection algorithm^59^ and based on their between affinities cell clusters were collapsed to 7 distinct populations corresponding to discrete developmental stages. Developmental pseudotime and transitions between populations were inferred based on RNA velocity (Fig. 6d and Supplemental Fig. 18a–c). Overall, we found the populations to be highly homogeneous in terms of ex vivo culture sample composition. Not unexpectedly, we observed higher sample admixture in populations corresponding to early time-points, consistent with the notion that cells at this stage had yet to establish lineage fate (Supplementary Fig. 18d).

**Fig. 7:**
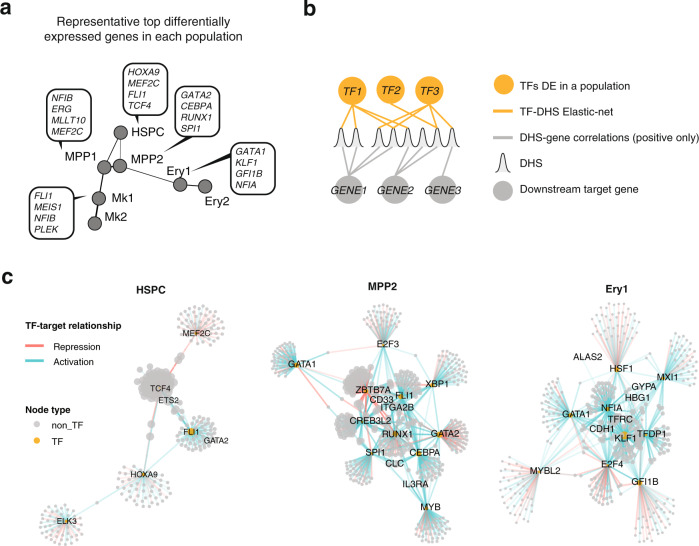
Defining the stage-specific erythroid regulatory networks. **a** Representative top differentially expressed TF genes between populations as identified by Wilcoxon sum rank test. **b** Schematic diagram of the regulatory network construction logic. Differentially expressed TFs among single-cell populations are assigned to their target genes based on elastic-net regression results. DHSs are linked to their target genes using correlation between population level DHS density and gene expression. **c** Regulatory networks initiated by TFs specific to each population. Up to top 50 target genes are shown for each TF. Transcription factors and select marker genes are annotated.

Using lineage trajectories inferred from transcriptional transitions between populations, we identified two major pathways starting from a cluster with HSPC signature (HSPC cluster) leading to terminal megakaryocytic and erythroid fates (Fig. 6c). Transitions from HSPC cluster to lineage specific clusters involves two clusters with progenitor gene signature (MPP1, and MPP2) each of them stemming from the HSPC cluster. MPP1 consists primarily of cells sampled from megakaryocytic cultures while ~25% of the cells in the population are derived from day 2 of the erythroid differentiation. MPP1 maintains a broader early progenitor signature (Fig. 6e and Supplementary Fig. 18e, f) and transitions to a population with early Mk signature (Mk1) which eventually gives rise to mature megakaryocytic population (Mk2). MPP2 is composed almost exclusively of early (day 2 and day 4) erythroid cells and appears as a nodal cluster with affinities to both the early erythroid cluster as well as the Mk primed MPP1. Importantly, MPP2 exhibits gene expression signature characteristic of various myeloid subtypes (Fig. 6e and Supplemental Fig. 18e, f) expressing critical myeloid regulators alongside megakaryocytic and erythroid ones (Supplementary Data 9). Stage-specific TF network reconstruction using TF-DHS and DHS-gene assignments (Fig. 7a, b), reveals that the myeloid signature is orchestrated by a core network of critical myeloid regulators (SPI1/PU.1, CEBPA, and FLI1), specific to MPP2 (Fig. 7c). This is compatible to single cell TF protein dynamics during ex vivo erythropoiesis, demonstrating that multiple TFs of alternate hematopoietic lineages are active in early progenitors prior to emergence of CFU-e populations^60^. Furthermore, early erythroid progenitors display affinities with myeloid and basophilic lineages transiently emerging in the culture. Here, in an attempt to identify the origin of this myeloid population present in our experiments, we performed FACS timecourse for the myeloid marker CD33 during both erythropoiesis and megakaryopoiesis. We find that the CD33^+^ population is a subset of CD34^+^ HSPCs as >80% of uncultured CD34^+^ are also positive for CD33. During erythroid differentiation, we find that cells transiently undergo a state of CD33^+^/CD117^+^, whereby day 6 the majority (~70%) has transitioned to CD33^-^/CD117^+^ (Supplementary Fig. 19). In contrast, expression patterns of CD33 and CD41 during ex vivo megakaryopoiesis are mutually exclusive, confirming the erythroid-specific origin of the myeloid population.

In order to compare our findings to steady-state hematopoiesis, we analyzed previously published single-cell RNAseq data from FACS fractionated BM-derived hematopoietic populations^61^. Trajectory inference on Force Atlas embedding using PAGA and DPT pseudotime analysis revealed two major differentiation pathways originating from a developmentally primitive population with HSPC. One with defined erythroid signature, and one exhibiting a myeloid gene expression profile (Supplementary Fig. 20a). Although we were able to detect a few cells with megakaryocytic signature concentrated close to HSPCs, their population is very small and no distinction between primitive and mature megakaryocytes could be made. Additionally, gene expression patterns of megakaryocytic markers and TFs are not well defined to infer differentiation trajectory (Supplementary Fig. 20a). Upon determination of cell clusters (Supplementary Fig. 20b) we detected a population of cells which expresses several myeloid markers and particularly those of basophils (e.g., *LMO4, CLC*), and appears to originate from two populations early on the erythroid trajectory (Supplementary Fig. 20b). In order to match the identified ex vivo populations to bone marrow steady states we integrated the two datasets through a *k-*nearest neighbor analysis (Supplementary Fig. 21). While HSPC and erythroid states exhibited extensive overlap between the two datasets, the ex vivo megakaryocytic populations displayed an ill-defined pattern given the limited representation of Mks in the bone marrow dataset. Interestingly, the MPP2 population exhibited strong affinity with the basophil population (Louvain cluster 12) as well as cells early along the erythroid and myeloid trajectories, confirming the myeloid origin of this population. In order to compare gene expression profiles along the erythroid trajectory inferred from either ex vivo differentiated erythroid cells or BM fractionated populations we correlated gene expression profiles from 1000 top highly expressed genes in both datasets. This revealed that ex vivo erythropoiesis recapitulates exceptionally well the gene expression dynamics from native erythroid populations with median Spearman’s *⍴* = 0.81 (Supplementary Fig. 22). These results further support our population-level findings about the transient emergence of a population during erythroid development that maintains myeloid capacity. The affinity between the basophilic lineage and the erythroid has been previously described^61,62^ and it has been suggested that basophils derive from erythro-myeloid progenitors^63^.

## Discussion

Here, we systematically link individual transcription factors and their target *cis*-elements along ex vivo human erythropoiesis, resolving how these elements organize temporally, encoding lineage commitment and differentiation during hematopoiesis. More recently, multiple efforts have extensively studied the individual (*cis*- and *trans*-) regulatory components involved in erythropoiesis^22^ as well as other diverse hematopoietic lineages^9,64–67^. The bulk of these efforts however base their findings either on immunophenotypically defined hematopoietic populations, or single-cell dissection of steady state heterogeneous sources, where developmental relationships between cells within a heterogeneous steady-state population can only be inferred^13,15,61,68^. In this work we overcome the limitations associated with immunophenotypic isolation of hematopoietic populations^57,69^ which often fail to capture transient or rare populations, while enrichment for specific populations is entirely dependent on the immunophenotypic panel used for fractionation^70^. By capitalizing on the continuity of the differentiating populations during ex vivo erythropoiesis we finely map chromatin accessibility and gene expression dynamics enabling the direct and repeated measurement of the dynamic epigenetic landscape along a defined lineage trajectory. In addition, a dense sampling approach enables the unbiased detection of transient events occurring over short intervals that would otherwise be missed by sparse sampling methodologies.

Through integrative analysis of chromatin accessibility and gene expression during erythropoiesis we draw thousands of links between individual distal regulatory elements and their target genes at much higher resolution than that afforded by other methods. This approach revealed a sequence of discrete, non-overlapping regulatory modules comprising of interacting transcription factors and individual *cis*-regulatory elements, corresponding to distinct stages of erythroid development. Strikingly, the transition between the activity of these modules coincides with a sequence of experimentally validated rapid lineage restriction events. We found that the exit from the program associated with the HSPC state occurred independently of lineage outcome, as it was also identified during ex vivo megakaryopoiesis. Moreover, comparison of developmental transcriptomics of single cells along erythropoiesis and megakaryopoiesis reveals that exit from HSPC occurs over the same developmental interval for both lineages, indicative of a mechanism independent of the cytokine environment. This finding adds to previous reports that activation of murine bone marrow HSCs with different lineage cytokines induces a common repression mechanism of HSC signature while activates genes implicated in differentiation in a cytokine independent fashion^71^.

Upon exit from the HSPC state we found the two lineages to exhibit differential commitment kinetics. Erythroid differentiation maintains a broader myeloid lineage capacity (Ery, G/M, Mk) prior to erythroid commitment as a result of a transient upregulation of a regulatory program involving canonical myeloid transcription factors (FLI1, SPI1, C/EBPs, GATA2, etc.). Network analysis in the progenitor stage prior to erythroid commitment, demonstrates that FLI1 is a central TF with extensive affinities to other transcriptional regulators, ultimately gatekeeping the fate choice between the megakaryocytic and erythroid lineage. There are several lines of evidence from single-cell assays in both mouse and human hematopoiesis suggesting that erythroid, megakaryocytic and basophilic lineages emerge from a shared population^13,16,60,64,72,73^, while mass cytometry dynamics of lineage-specific transcription factors ascribe FLI1 the role of “gatekeeper” between the erythroid and the megakaryocytic fate^60^. The findings presented here, however, demonstrate that this lineage-permissive transcriptional program is restricted only to erythropoiesis. This is compatible with previous experimental evidence demonstrating the affinity of basophilic lineage to the erythroid branch^61^, specifically. Additionally, results from transgenic mice lacking a set of the C/EBP family of myeloid regulators that exhibit decreased erythroid output^74^. In contrast, transcriptional, functional and phenotypic evidence from ex vivo megakaryopoiesis presented here, suggest rapid megakaryocytic commitment upon HSPC exit. These results align well with the growing evidence suggesting that megakaryocytic commitment is occurring earlier compared to erythroid fate^75^ and that megakaryocytic lineage arises directly from the primitive hematopoietic compartments^57,76–78^.

In order to reconcile our findings on lineage commitment from bulk populations with transcriptional dynamics from individual cells we compiled one of the most comprehensive analyses of single-cell gene expression along a closely monitored developmental system, so far. Furthermore, this dataset represents the first, to our knowledge, single-cell description of gene expression dynamics along the ex vivo megakaryocytic development from purified HSPCs. Although different approaches have been suggested to enrich for megakaryocyte and platelet biased progenitors and dissection of the bipotent MEPs^79–81^ there is no consensus purification scheme to isolate cells at different stages of megakaryocytic development with the resolution available for erythroid development^82,83^. This is primarily due to the rarity of Mk cells in the bone marrow^84,85^ and the fragility of the mature large endomitotic megakaryocytes. Here, however, we present an unbiased global view of gene expression and lineage commitment dynamics of megakaryocytic development based on equiproportional sampling of populations along megakaryopoiesis.

Overlaying the information of sampling timepoint of each population allowed us to match shifts in TF expression in single-cells to the regulatory programs identified from our population-level experiments. Strikingly, our single-cell based observations recapitulate both our ex vivo population-based findings as well as single-cell transcriptional dynamics from bone marrow fractionated populations with remarkable fidelity. This suggests that highly synchronized rapid shifts in gene expression levels of lineage regulators across individual cells, occurring over short intervals of developmental time, underpin the changes observed in bulk populations. This contrasts the current sentiment hinging on observations from single-cell analyses where variability in the chromatin and transcriptional landscapes among steady-state populations are interpreted as gradients of continuous regulatory states^13,14,68,86^.

Here we present insights into the developmental regulatory dynamics during hematopoiesis illuminating mechanisms of lineage commitment, previously obscured by sampling biases and limitations. Although we draw parallels with steady-state in vivo derived data, we appreciate that the artificial nature of the ex vivo culture systems can be a confounding factor. Nevertheless, we highlight the utility of ex vivo development systems in studying rare or otherwise inaccessible populations in vivo and provide a generalizable framework of how interactions between the *trans*-environment and the chromatin instruct fate choice and lineage commitment during development. Additionally, the dense sampling and the systematic linkage between distal elements and target promoters results in high-resolution charts of the stage-specific activity of regulatory elements. Beyond the standpoint of gene regulatory mechanisms, such elements can prove particularly useful in transgene-based therapies where the efficacy of these methods relies on the precise modulation of gene expression in a developmental and lineage-specific manner.

## Methods

### Ex vivo erythropoiesis

For the ex vivo induction of erythroid and megakaryocytic differentiation, human CD34^+^ enriched PBMC ( > 90% purity) from 3 different GCSF-mobilized healthy adult donors (Fred Hutchinson Cancer Research Center—Cooperative Centers of Excellence in Hematology Core B) were used. Cells were isolated under the protocol approved by the Fred Hutch Institutional Review Board (protocol no. 985.03) and in accordance with the Declaration of Helsinki. Prior to culture, cells were thawed rapidly in a 37 °C water bath and cultured overnight in recovery media containing IMDM, supplemented with StemSpanTM CC110 (Stemcell Technologies). For erythroid differentiation an established 3-step differentiation scheme was used^23^. Briefly, CD34^+^ cells were cultured for 7 days in IMDM media containing 0.1 µg/mL rhSCF (PeproTech), 0.005 µg/mL rhIL-3 (PeproTech), 3 U/mL rhEPO (PeproTech), 5% human AB plasma, 2 U/mL heparin, 0.01 mg/mL Insulin, 0.33 mg/mL holo-transferrin (Millipore-Sigma), 1 µM hydrocortisone (Millipore-Sigma), 1x Penicillin/Streptomycin (ThermoFisher Scientific), followed by culturing in the same media for 4 more days without IL-3 and hydrocortisone for 4 days.

### Ex vivo megakaryopoiesis

CD34 + HSPCs were differentiated to the megakaryocyte lineage by culturing for 11 days in IMDM based media containing 30 ng/mL rhTPO (PeproTech), 1 ng/mL rhSCF (PeproTech), 7.5 ng/mL rhIL-6 (PeproTech), 13.5 ng/mL rhIL-9 (PeproTech), 20% BIT supplement (StemCell Technologies), 40 µg/mL LDL (Millipore-Sigma), 0.05 mM beta mercaptoethanol (Millipore-Sigma), and 1x Penicillin/Streptomycin (ThermoFisher Scientific).

### HUDEP-2 expansion

HUDEP-2 cells were kindly provided Ryo Kurita and Yukio Nakamura, Cell Engineering Division, RIKEN BioResource Center, Tsukuba, Ibaraki, Japan. HUDEP-2 cell culture HUDEP-2 cells were maintained in StemSpan H3000 medium (Stem Cell Technologies) supplemented with 100 ng/ml hSCF (Peprotech), 3 IU/ml erythropoietin (Peprotech), 10^-6^ M dexamethasone (Millipore Sigma) and 1 mg/ml doxycycline (Millipore Sigma).

### Genetic knock-outs in HUDEP-2 cells

All TALEN pairs were assembled according to established protocols^38,87^. Sequences of the TALEN binding domains are listed in Supplementary Table 1. In vitro transcription of TALEN-mRNA was performed by the T7 mScript™ Standard mRNA Production System (CELLSCRIPT, C-MSC100625), including 5’-capping and poly-A addition reactions. Electroporation was performed as previously described^88^. Briefly, 2 μg of each TALEN monomer mRNA were transfected in HUDEP-2 cells using a BTX electroporator (ECM 830, Holliston, MA) in 100 µL of BTX Express electroporation solution. Double knock out clones were detected post electroporation and single cell sorting (﻿MoFlo Astrios, Beckman Coulter) by Out-Out (CDH1 promoter and HS1) or In-Out PCR (HS2). PCR primer sequences are provided in Supplementary Table 2.

### Colony forming assays

Colony forming assays were performed by sampling ~1000 cells daily from either erythroid or megakaryocytic primary cultures from 2 donors and plating each in duplicates in 35 mm dishes containing MethoCult H4435 (StemCell Technologies). After two weeks, colonies were identified and scored under a dissection microscope.

### Suspension lineage potential assays

For megakaryocytic potential assays, ~100,000 cells were sampled daily from erythroid cultures until day 7. After removing primary media, cells were transferred to secondary suspension cultures containing megakaryocytic media as described above and allowed to grow for two weeks. Megakaryocytic potential was estimated by the frequency of CD41^+^ cells with flow cytometry on day 12 of the secondary megakaryocytic culture. Similarly, for erythroid potential assays during megakaryocytic differentiation, ~100,000 cells were sampled daily until day 7 from primary megakaryocytic differentiation and transferred to secondary erythroid media. After two weeks in the secondary media, erythroid potential was assessed by the frequency of CD235a^+^ cells present with flow cytometry.

### Flow cytometry

Approximately 100,000 cells each day per culture replicate were harvested. Media were removed by washing with staining buffer (PBS + 0.25% BSA) and cells were labeled according to product specifications, incubating at 4 °C. Cells from erythroid differentiation were stained daily for CD117 PE at 1:10 dilution (Cat. No.: 340529, Clone 104D2, BD Biosciences), and CD235a at 1:20 dilution (Cat. No.: 559943, Clone GA-R2/HIR2, BD Biosciences). Megakaryocytic differentiation was monitored by staining with CD41 PE at 1:10 dilution (Cat. No.: 555467, Clone HIP8, BD Biosciences), and CD42b APC at 1:10 dilution (Cat. No.: 551061, Clone HIP1, BD Biosciences). Megakaryocytic potential of primary erythroid cultures was determined by measuring CD41a PE at 1:10 dilution (Cat. No.: 555467, Clone HIP8, BD Biosciences) expression in the secondary cultures. Myeloid populations were identified by CD33 expression staining with Alexa Fluor 700 anti-human CD33 at 1:20 dilution (Cat. No.: 561160, Clone WM53, BD Biosciences). For detection of genetic knock-out of CDH1 in HUDEP-2, APC anti-human CD324 at 1:20 dilution (E-Cadherin) (Cat. No.: 324108, Clone 67A4, Biolegend) was used. Samples were acquired on CytoFlex S (Beckman Coulter) and analyzed using FlowJo (Becton Dickinson). All analyses were performed on the live, single events as determined by their profile on the FSC-H, FSC-A, and SSC-A.

### DNase I accessibility

Erythroid cultures from 3 donors were sampled on a daily basis from day 0 to day 12 and >100,000 cells were harvested per DNase I reaction adapting the protocol from John et al.^89^. After removing media washing twice with ice-cold PBS and centrifugation for 5 min at 400 × *g*, cells were washed with ice-cold Buffer A (15 mM Tris-HCl pH 8.0, 15 mM NaCl, 60 mM KCl, 1 mM EDTA pH 8.0, 0.5 mM EGTA pH 8.0, 0.5 mM spermidine). Cells were resuspended in ice-cold Buffer A and equal volume of lysis buffer (0.04% of IGEPAL CA-630 de-ionized with AG^®^ 501-X8(D) Mixed Bed Resin, cat:1436425 Bio-Rad, in Buffer A) was added and cells were incubated at 4 °C for 10 min. Nuclei were then moved to 37 °C and replicates of each sample were incubated with a gradient of DNase I (D4527, Sigma-Aldrich) solution (ranging from 40 IU to 100 IU of DNase I) of equal volume and the reaction was allowed to proceed for 3 min at 37 °C. DNase I digestion was quenched by adding a volume of 5X Stop buffer (50 mM Tris-HCl pH 8.0, 100 mM NaCl, 0.1% SDS, 100 mM EDTA pH 8.0) equal to the volume of cell suspension (100 µL final reaction volume) and the reactions were incubated at 37 °C for 60 min. After incubation, 1 µL of Proteinase K (Sigma) was added to each reaction followed by incubation at 50 °C for 60 min. Digested genomic DNA was visualized on 1.2% agarose gel and the fragment size profile was generated using the Fragment Analyzer (Advanced Analytical).

Prior to genomic library generation, fragments were subjected to size fractionation. Large DNA fragments were removed mixing the DNase I digested sample with a Polyethylene-Glycol (PEG 8000, Sigma) solution containing 8.3 mg of carboxylate-modified magnetic particles (Sera-mag beads, Thermo) to a final PEG concentration of 6% w/v. Sample was incubated at room temperature with constant mixing. Bead-bound fragments were removed by magnetic separation and a PEG solution (39.5% w/v final) containing 7.15 mg magnetic beads was added to the supernatant. After >90 min incubation at room temperature with constant mixing, the supernatant is removed by magnetic separation and discarded while bound DNA fragments are eluted from the beads. The eluate is then subjected to a second binding by mixing with a PEG solution (38.5% w/v final) containing 7.15 mg of magnetic beads. The solution is incubated at room temperature for >90 min with constant mixing. The supernatant is removed by magnetic separation and the fragments are eluted from the beads. Fragment size distribution and concentration of the fractionated sample was measured with Fragment Analyzer (Advanced Analytical). Illumina compatible, double-stranded DNA library libraries from the size fractionated samples were constructed using the ThruPLEX DNA-seq Kit (Takara Bio) according to manufacturer’s instructions. DNase I-seq libraries were sequenced on NextSeq 500 (Illumina) with a 2x36bp read length. Adapter trimmed FASTQ files were aligned against GRCh38 using the BWA aligner^90^. All downstream DNase I-seq analyses were performed on DNase I hotspots (genomic regions with statistically significant enrichment in DNase I cleavage)^31,91^. Hotspots were detected using hotspot2 program (https://github.com/Altius/hotspot2).

### Total RNA sequencing

For gene expression analysis, total RNA was collected using the mirVana RNA isolation kit (ThermoFisher Scientific) or RNeasy Mini Kit (Qiagen) from sorted (>20,000 cells) and bulk cultures (>1,000,000 cells). Illumina libraries were constructed using the TruSeq Stranded Total RNA with Ribo-Zero Globin (Illumina). Finally, libraries were quantified using Fragment Analyzer (Advanced Analytical). RNA-seq libraries were sequenced with HiSeq 4000 (Illumina) using a 2x76bp read length and alignment was performed using STAR Aligner^92^ against the GRCh38 reference genome. Gene counts were obtained using featureCounts^93^ and FPKM per gene were calculated using Cufflinks^94^. Values were normalized using quantile normalization.

### Identification of developmentally regulated DHSs and genes

To identify developmental responses in chromatin accessibility and gene expression, we employed a regression analysis strategy. A robust linear regression was performed on the quantile-normalized, mean-centered DHS density values or gene counts from 3 donors by fitting a cubic spline with 3 degrees of freedom using the lmrob function from the robustbase R package and fitted values were recorded. Statistical significance was estimated by performing a likelihood ratio test against a null model where developmental time was removed as a term. Likelihood ratio test (LTR) *p*-values were adjusted for false discovery rate (FDR) using the Benjamini–Hochberg method. A refined list of the statistically significant changing DHS was obtained by setting the maximum normalized DHS density to ≥30 counts and log_2_-fold difference between the minimum and maximum daily average values to 1. Significantly changing genes were further filtered by excluding genes with maximum FPKM below 2 and those with log_2_-fold difference between the minimum and maximum daily average gene counts below 1.

### Identification of TADs and interaction loops from Hi-C data

Capture Hi-C data from primary CD34^+^ hematopoietic progenitor cells were obtained from Misfud et al.^29^. Ex vivo differentiated day 11 erythroid progenitors derived Hi-C data were obtained from Huang et al.^30^. Reads were processed with pairtools (https://github.com/open2c/pairtools) removing reads with MAPQ < 30. Contact matrices were generated with Cooler^95^. Topologically associated domains (TADs) were computed with HiCExplorer^96^ on KR balanced matrices at 10 kb resolution. Chromatin interaction loops were predicted using Mustache^97^ from the KR balanced matrices at 10 kb resolution.

### Transcription factor recognition sequence enrichments

A list of transcription factor motif position weight matrices (PWM) was compiled from JASPAR 2018^98^ and HT-SELEX derived human transcription factor binding specificity models^99^. Motifs were then scanned across the GRCh38 reference genome using FIMO^100^ and motifs with nominal *p*-value <10^−4^ were mapped to DHS using BEDOPS^101^. Over- or under-representation of transcription factor motif clusters in each of the K-means DHS clusters was tested by performing a one-tailed hypergeometric enrichment test. *P*-values were adjusted using the Benjamini–Hochberg method^102^.

### Elastic net regression and regulatory module identification

To identify the transcription factors that modulate the accessibility of DHS a two-step regression with elastic-net regularization was utilized. Specifically, the per-day averaged accessibility of each DHS over time was expressed as a function of the per-day averaged expression of the transcription factors with motifs found in the DHS. We considered 214 transcription factors which had maximum expression >2 FPKM and for which there was available binding motif information. An elastic net support vector machine was initially trained on a subset of the data by omitting 4 timepoints using the glmnet R package. Optimization for the mixing parameter *α* (0 < *α* < 1) and the penalty stringency parameter λ was performed with a 100-fold cross-validation of each seat of *α* (ranging from 0 to 1 with 0.01 increments) and *λ* (100 equiproportional values ranging from 10^–5^ to 10^5^) parameters using the cv.glmnet function. Mean squared error (MSE) and standard error (SE) for each parameter pair tested and each DHS were recorded. Given that each DHS cluster is characterized by specific features (i.e., DHS density profile, sequence composition, and DHS overlap with other tissues) we sought to optimize elastic net parameters for each cluster, rather than overfitting each DHS individually. Therefore, for each cluster of DHS, the pair of *α* and *λ* parameters which minimized the total MSE and SE for that cluster was selected and subsequently supplied into a final elastic net regression which was applied to each DHS in a cluster (E1-E5). The capacity of elastic-net TF coefficients to accurately classify the DHS into their respective *K*-means cluster (E1-E5) against the TF motif counts was evaluated using a naïve Bayes classifier using fastNaiveBayes (https://github.com/mskogholt/fastNaiveBayes). Discrete motif counts per DHS for TFs with at least one non-zero elastic-net coefficient (192 out of 214) were modeled by fitting a Poisson distribution while elastic-net coefficients for the same set of TFs were modeled with Gaussian fit and prediction accuracy was calculated as the percent of correct classifications. Transcription factors with >200 DHS positively associated (52 out of 214 TFs) were taken into consideration and a hierarchical clustering on the cosine distances of the regression coefficients was performed. Cutting the tree at the 5 highest order clades using *k* = 5 resulted in 5 clusters of transcription factors which together with their associated DHS constitute a regulatory module.

### Single-cell RNA sequencing and data processing

Erythroid and megakaryocytic cultures were induced from the same donor as described above and on days 2, 4, 6, 8, and 11 cells were harvested and stored in liquid nitrogen using CryoStor CS10 (StemCell Technologies) until library preparation. On the day of library preparation, frozen cultured cells as well as an uncultured vial of CD34^+^ cells from the same donor were simultaneously processed. Cells were prepared for library preparation according to manufacturer’s instructions using the Chromium Single Cell 3′ Reagent Kit, version 1 (10X Genomics) for CD34^+^ and erythroid samples, except day 2. Chromium Single Cell 3′ Reagent Kit, version 2 (10X Genomics) was used for all megakaryocytic differentiation time points, and erythroid day 2. All libraries were sequenced on HiSeq 4000 (Illumina). Raw sequencing data were processed using Cell Ranger analysis pipeline v2.1.1. Reads were aligned against the hg19 reference genome.

### Single-cell RNA sequencing analysis of samples from primary erythroid and megakaryocytic differentiation

RNA velocity was computed using the CLI component of velocyto^103^ and Loom files with normalized transcript counts for each sample were generated with the ‘run10x’ command. Trajectory and pseudotime analysis was performed using scVelo^104^ and SCANPY^105^. Gene count matrix was filtered to include the top 2000 variable genes. Moments of spliced and unspliced kinetics were calculated using the first 30 components and 30 nearest neighbors. Cells were clustered using Leiden community detection algorithm^59^ and were further collapsed into biologically relevant populations. Cell connectivities, transitions, and pseudotime were computed based on RNA velocity. Developmental trajectories were inferred using PAGA^106^ on velocity connectivities. Transitions were projected on a Force-Atlas (FA) graph embedding using a velocity pseudotime prior. Marker genes per population were identified using Wilcoxon-rank test for every population versus its immediately connected populations. Significance was called on FDR-corrected *p*-values < 10^−5^ and absolute log_2_ fold-change ≥1. Overrepresentation of gene sets characteristic of specific cell-types, present within the marker genes from each population, was performed using ENRICHR^107^ available at (https://maayanlab.cloud/Enrichr/) using annotated gene-sets from the Human Gene Atlas database.

### Reconstruction of stage-specific transcription factor networks

For every differentially expressed transcription factor gene in each population, linked DHSs were called based on the elastic-net results. Subsequently, downstream target genes were identified based on positively correlated (Pearson’s *r* ≥ 0.7) DHS and genes. Networks were constructed using the igraph package (https://igraph.org/r/). Edge weights were calculated by standardizing the product of the elastic net coefficient and the pearson correlation value. For each node the centrality value was calculated. Network plots were generated using tidygraph (https://github.com/thomasp85/tidygraph) and ggnet2 (https://briatte.github.io/ggnet/) using the “fr” layout option.

### Single-cell analysis of human bone marrow derived data

Raw transcript count matrix obtained from Pellin et al.^61^ was filtered to include genes above 800 total counts and normalized to read depth and highly variable genes with minimum dispersion of 0.5 were selected. Trajectory and pseudotime analysis were performed using SCANPY as described above. Neighbors were computed on the first 30 principal components setting the number of neighbors to 30 and a Force-Atlas graph was computed. Cells were clustered using Leiden setting the resolution to 1.4. Diffusion pseudotime was calculated and cell-to-cell connectivities were computed using PAGA and projected on FA layout. Projection of single cell populations generated in this work on the bone marrow derived dataset was performed using the function “FindNeighbors” from the Seurat^108^ package with 20 neighbors returned (*k* = 20), after L2 normalization of the input data.

### Reporting summary

Further information on research design is available in the Nature Research Reporting Summary linked to this article.

## Supplementary information

Supplementary Information

Reporting Summary

Description of Additional Supplementary Files

Supplementary Dataset 1-9

## Data Availability

All DNase I and RNA sequencing data have been deposited to GEO under the series accession GSE182816. All processed data files available herein and relevant metadata are available on Zenodo [10.5281/zenodo.5291737]. Adult erythroid Hi-C data was downloaded from GEO, series GSE102201. Adult CD43 + HSPC Hi-C data obtained from ENA, accession ERR436024. Human bone marrow single-cell data was download from GEO using the following accessions: GSM3305359, GSM3305360, GSM3305361, GSM3305362, GSM3305363, GSM3305364, GSM3305365. Source data are provided with this paper.

## Source data

Source Data

## Code availability

All scripts and code used to process and analyze data herein are available upon request.
