# Supplementary material for: Discrete regulatory modules instruct hematopoietic lineage commitment and differentiation
Source: Nat Commun. 2021 Nov 23;12:6790. doi: 10.1038/s41467-021-27159-x (PMC8611072; doi:10.1038/s41467-021-27159-x)
Supplement: Supplementary file 1 — Supplementary Information [file 41467_2021_27159_MOESM1_ESM.pdf]

Supplementary information for

"Discrete regulatory modules instruct hematopoietic lineage commitment and differentiation"

Grigorios Georgolopoulos*, Nikoletta Psatha*, Mineo Iwata, Andrew Nishida, Tannishtha Som, Minas Yiangou, John A. Stamatoyannopoulos, Jeff Vierstra

Supplementary Figure 1

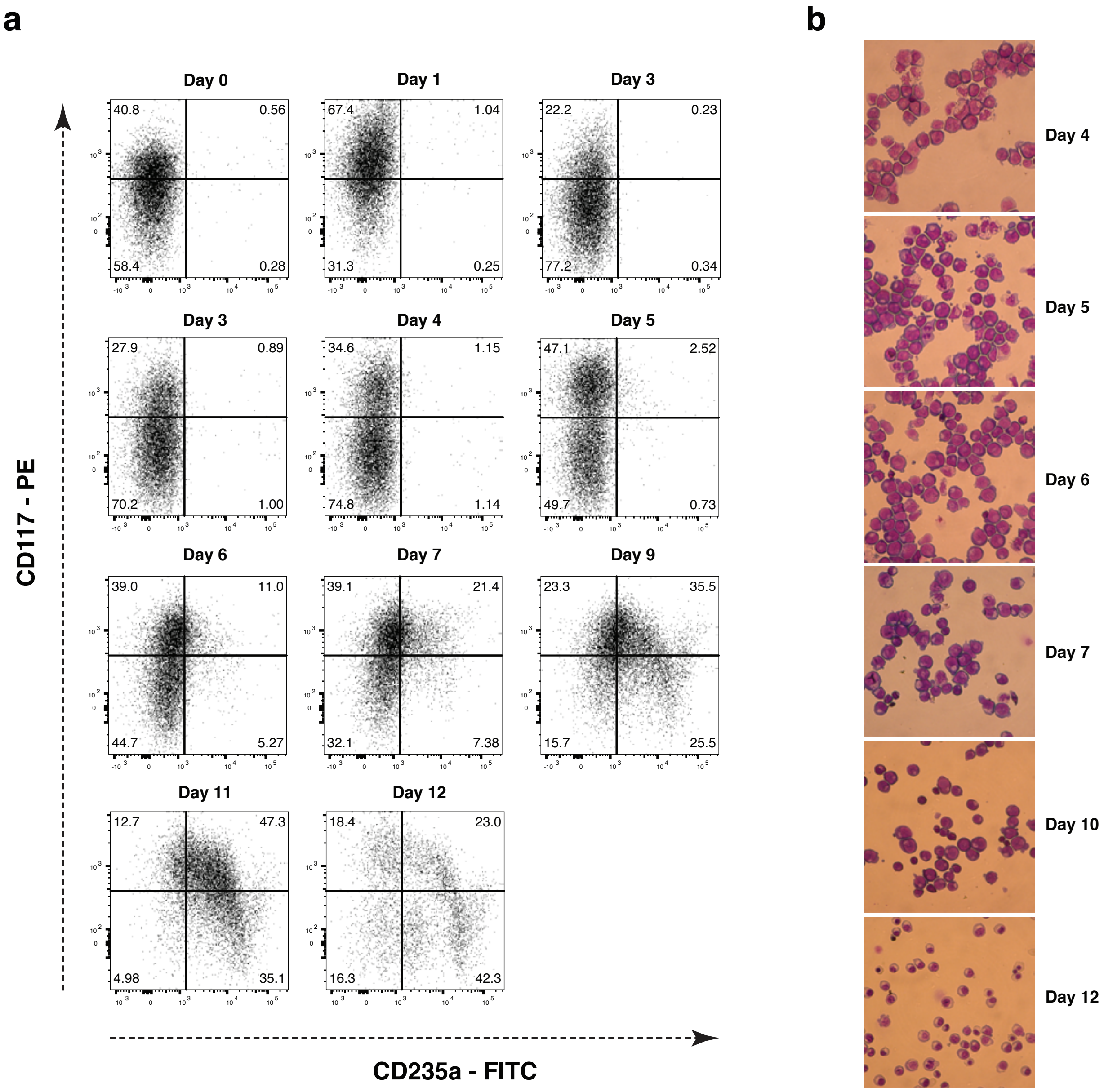

Supplementary Figure 1. Immunophenotypic and morphological characteristics of ex vivo erythropoiesis. (a) Flow cytometry time-course showing the expression profiles of two erythroid-specific cell surface markers, CD117 (C-Kit) and CD235a (Glycophorin A) over 12 days of culture. (b) Hematoxylin-Eosin staining of cytospin slides along the ex vivo erythropoiesis.

Supplementary Figure 2

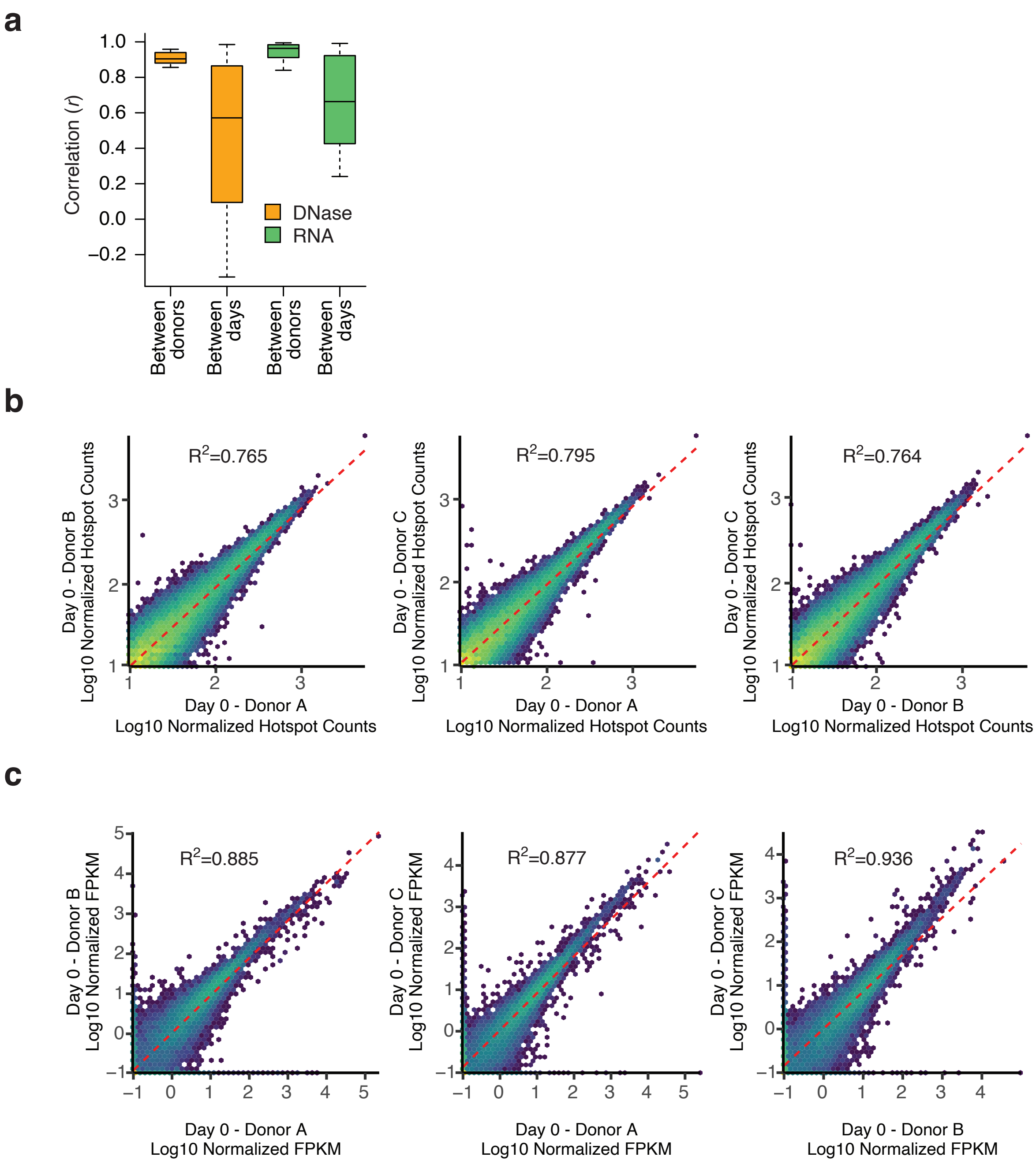

Supplementary Figure 2. Biological replicates are highly concordant. (a) Boxplots showing the correlation between donors and between days in DNase I-seq and RNA-seq experiments, respectively. Boxplot boundaries correspond to 25th and 75th percentile, respectively and median is shown. Boxplot whiskers correspond to 1.5 x inter-quartile range excluding outliers. (b) Scatterplots of log10 normalized Hotspot (DHS) counts from day 0 and R2 between donors. Points are binned values and are colored by the number of points in each bin. (c) Scatterplots of log10 normalized gene counts (FPKM) from day 0 and R2 between donors. Points are binned values and are colored by the number of points in each bin.

Supplementary Figure 3

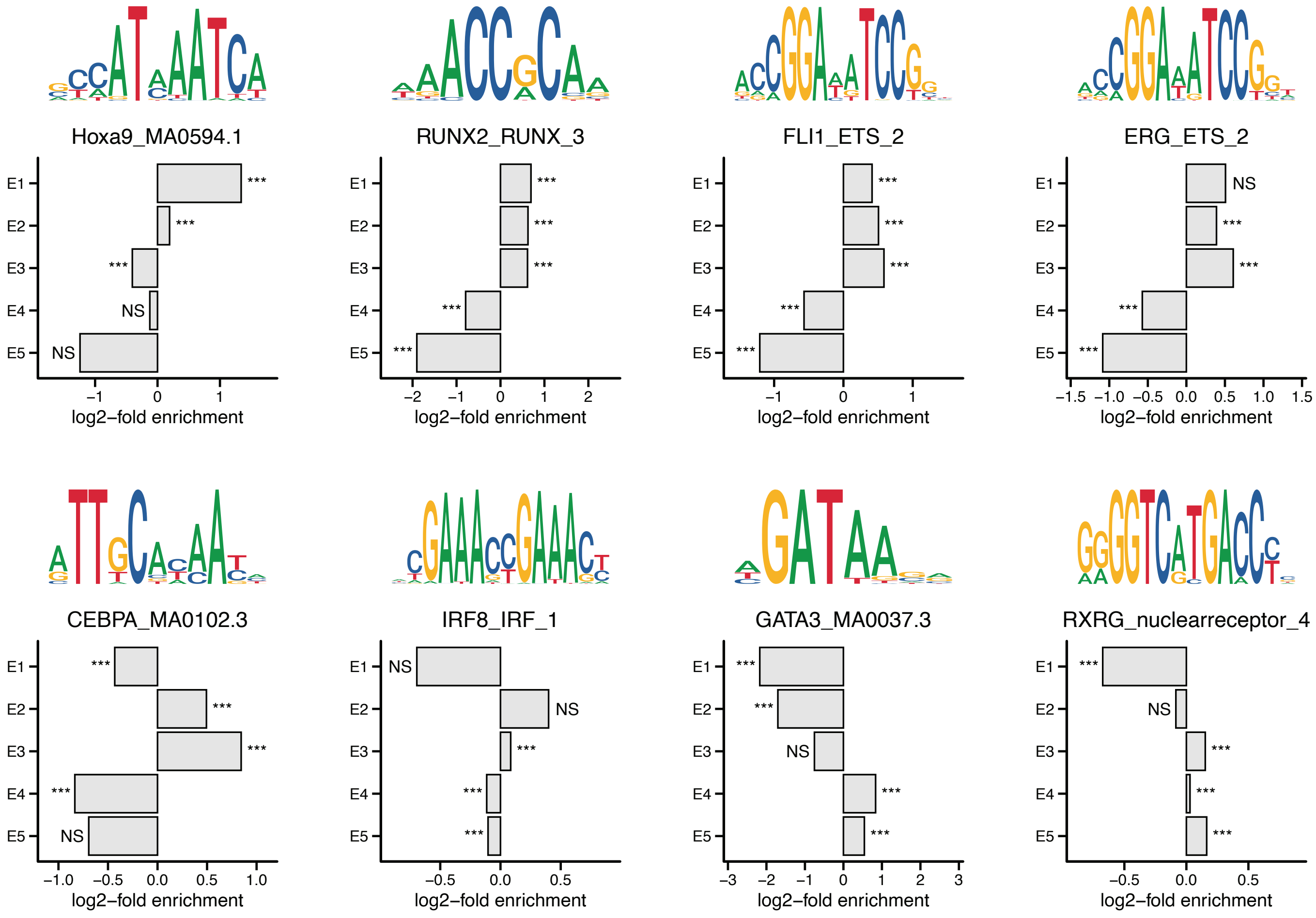

Supplementary Figure 3. Differential enrichment for transcription factor binding motifs across DHS clusters. Log2-fold enrichment (x-axis) for binding motifs of known regulators of hematopoiesis across the DHS clusters (E10-E5, y-axis). Triple asterisks indicate hypergeometric enrichment FDR < 0.05. N.S. indicates no significant enrichment.

Supplementary Figure 4

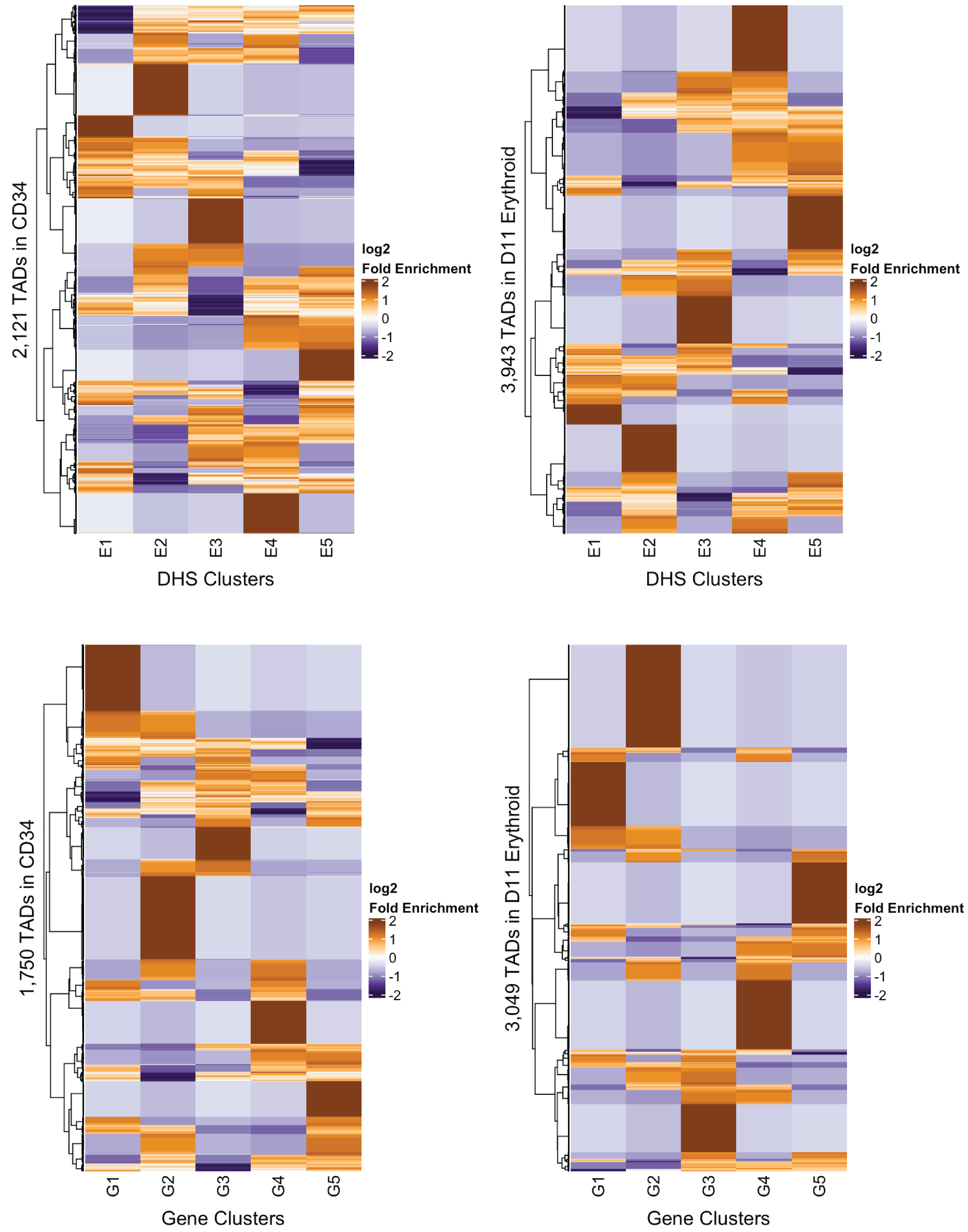

Supplementary Figure 4. Colocalization of developmentally co-regulated DHS and genes. Clustering of TADs identified in CD34+ and day 11 erythroid Hi-C data, respectively, exhibiting enrichment for DHS or genes from specific clusters.

Supplementary Figure 5

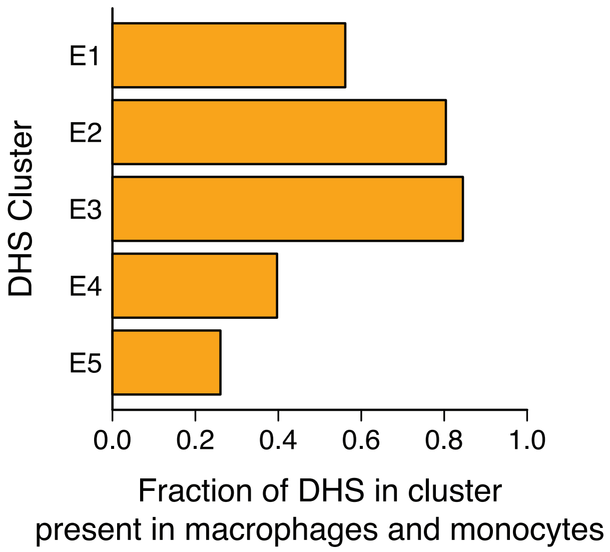

Supplementary Figure 5. DHS clusters E2 and E3 display an extensive shared chromatin landscape with other myeloid cell types. Percent of DHS in each cluster overlapping with DHS detected in CD14+ monocytes and macrophages.

Supplementary Figure 6

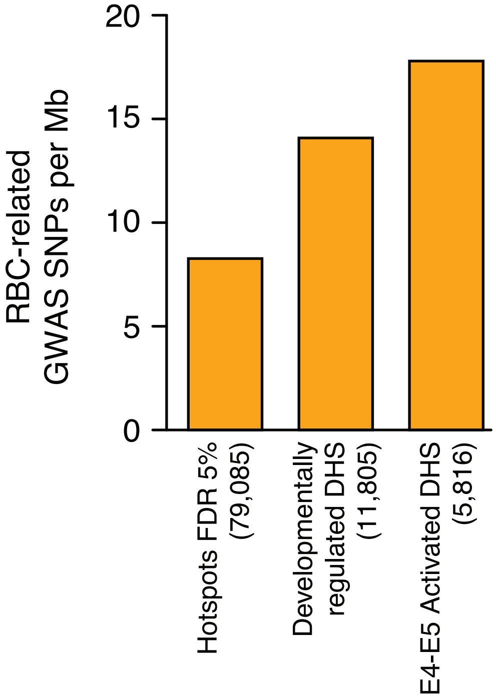

Supplementary Figure 6. Erythroid specific DHSs are enriched for GWAS traits related to clinical erythroid phenotypes. Frequency of GWAS SNPs associated with red blood cell (RBC) traits per Mb of all detected DHS (Hotspots FDR 5%), 11,805 developmentally regulated DHS and 5,816 late activated DHS from cluster E4 and E5.

Supplementary Figure 7

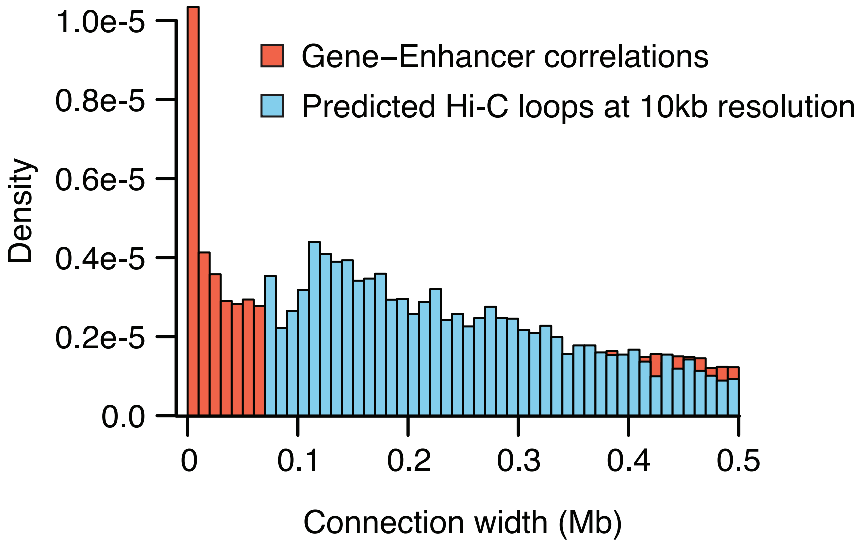

Supplementary Figure 7. Correlation based prediction of gene-enhancer connections identifies gene-proximal links. Density histogram of genomic distances (Mb) of correlation-based predictions of enhancer-gene links (red) and chromatin loops predicted from day 11 ex vivo differentiated erythroid progenitor Hi-C data (blue).

Supplementary Figure 8

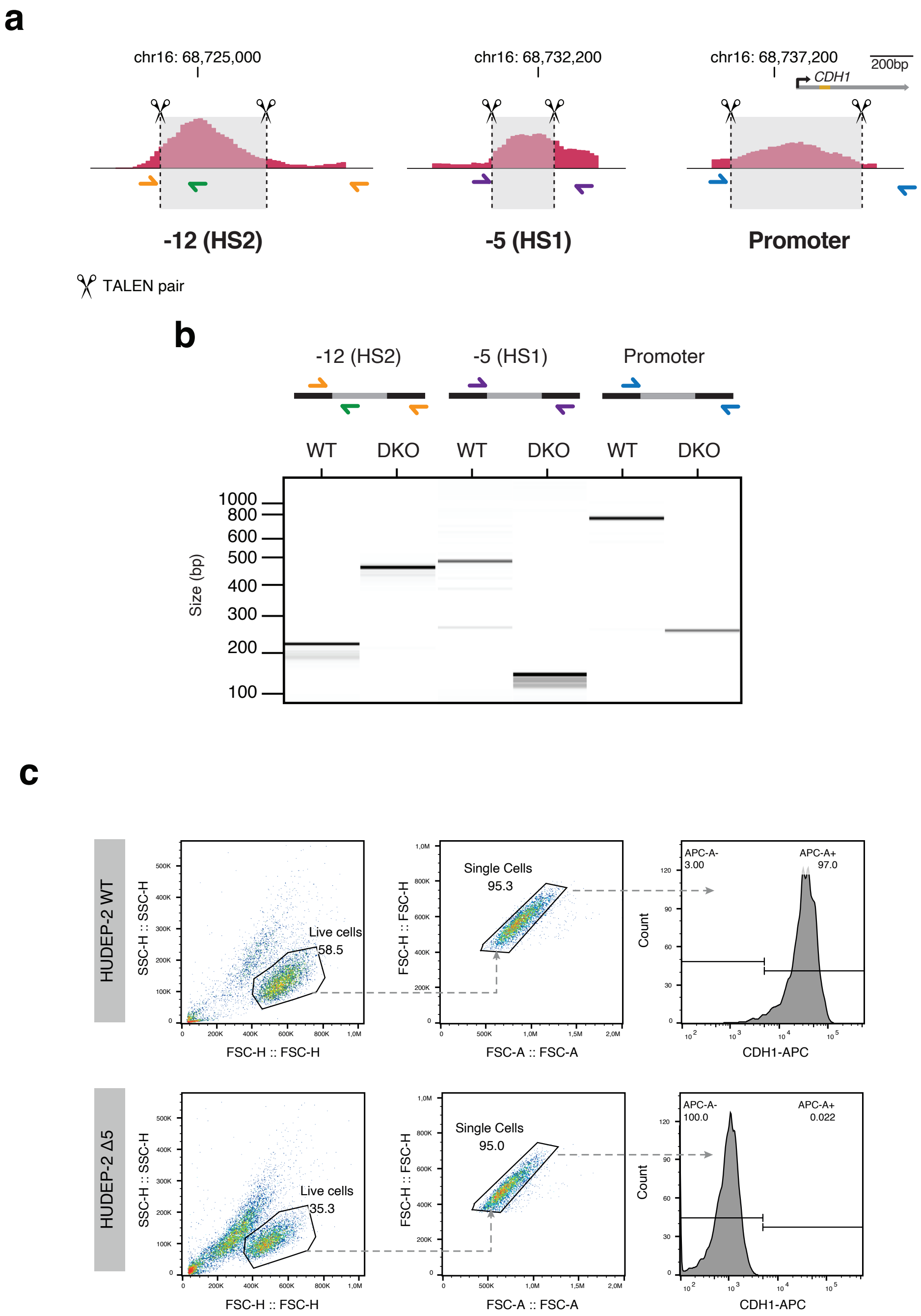

Supplementary Figure 8. Genetic knockouts confirm the predicted cis- regulators of CDH1. (a) Position of TALE-FokI nuclease pairs (stylized as scissors) and primers (colored half-arrows) used against each of the upstream DHS and the promoter. (b) PCR-based validation of the genetic knockouts in each region. In HS2 (-12) an “in-out” PCR approach was followed. Wild type amplifies from outside forward primer (left yellow arrow) and inside reverse (green arrow). HS2 knock-out removes the binding site of the inside reverse primer and amplifies from the two outside primers, resulting in a larger fragment than wild-type. (c) Representative gating strategy to determine CDH1 expression within the live, single events as determined by FSC and SSC.

Supplementary Figure 9

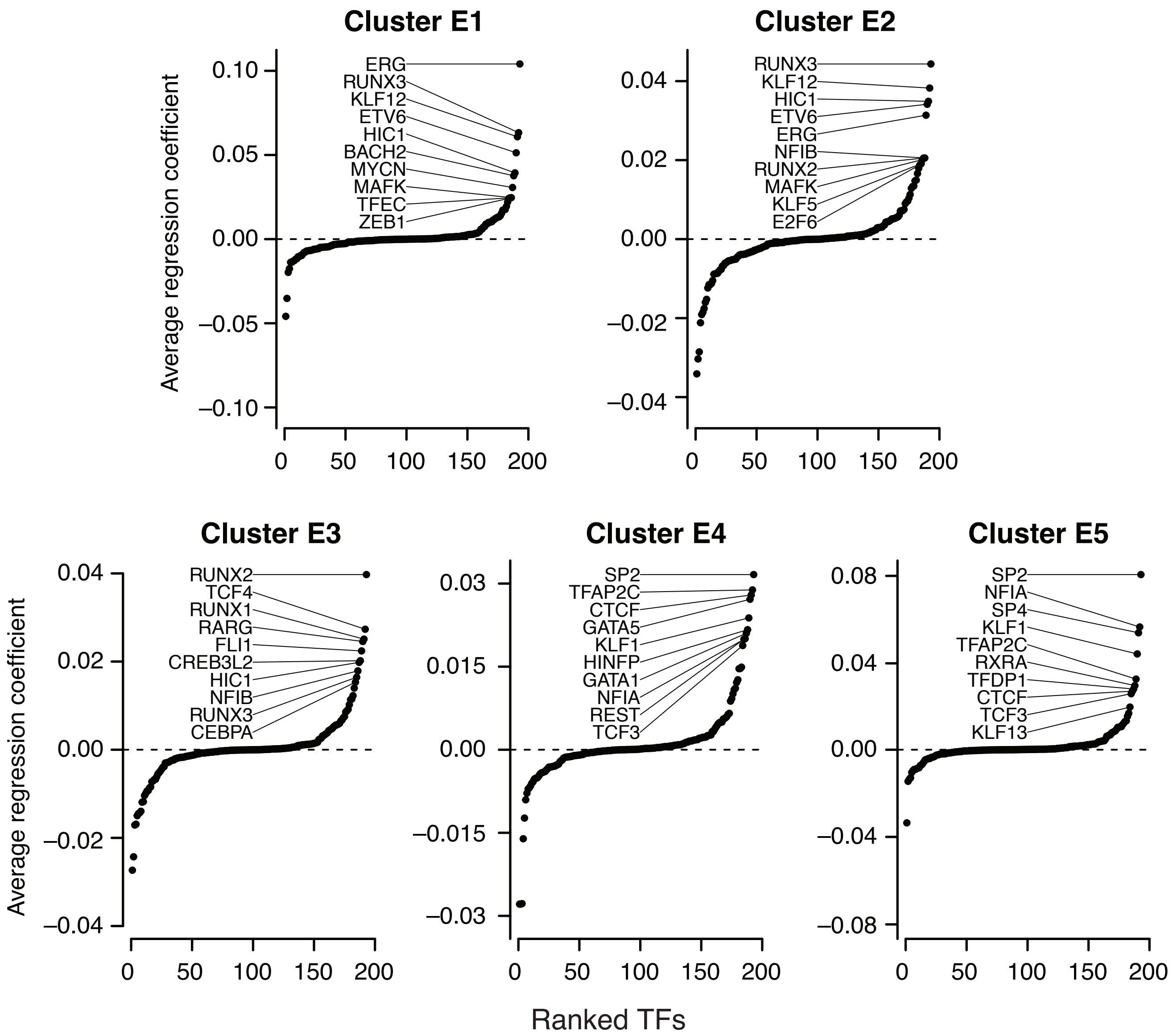

Supplementary Figure 9. Ranking TFs by average elastic-net regression coefficient per DHS highlights major trans- regulators during erythropoiesis. Ranked TFs based on the average regression coefficient across each DHS cluster (E1-E5).

Supplementary Figure 10

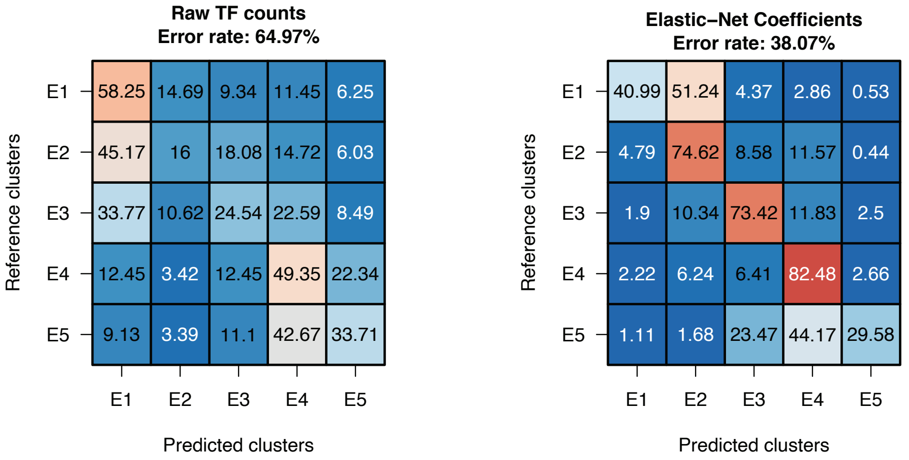

Supplementary Figure 10. Assessing the capacity of elastic-net TF coefficients and raw TF motif instances per DHS to predict the DHS cluster. Confusion matrices and overall error-rate of naïve Bayes classification of predicted DHS clusters using either discrete TF motif counts per DHS (top) or elastic net TF coefficients (bottom).

Supplementary Figure 11

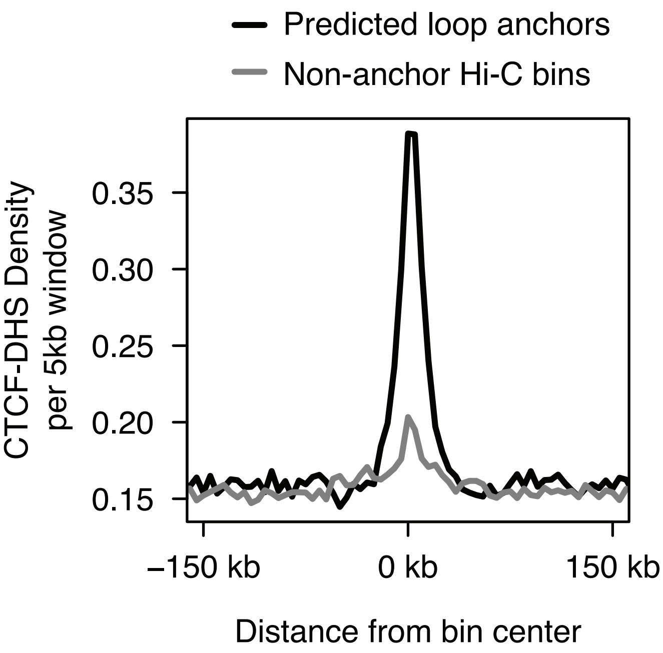

Supplementary Figure 11. Enrichment of CTCF bound DHS in predicted loop anchors. Density plot of DHS with > 1 CTCF motif in 5kb bins across a 300kb window around predicted loop anchors from ex vivo differentiated day 11 erythroid progenitor Hi-C data (black line) and permuted regions (grey line).

Supplementary Figure 12

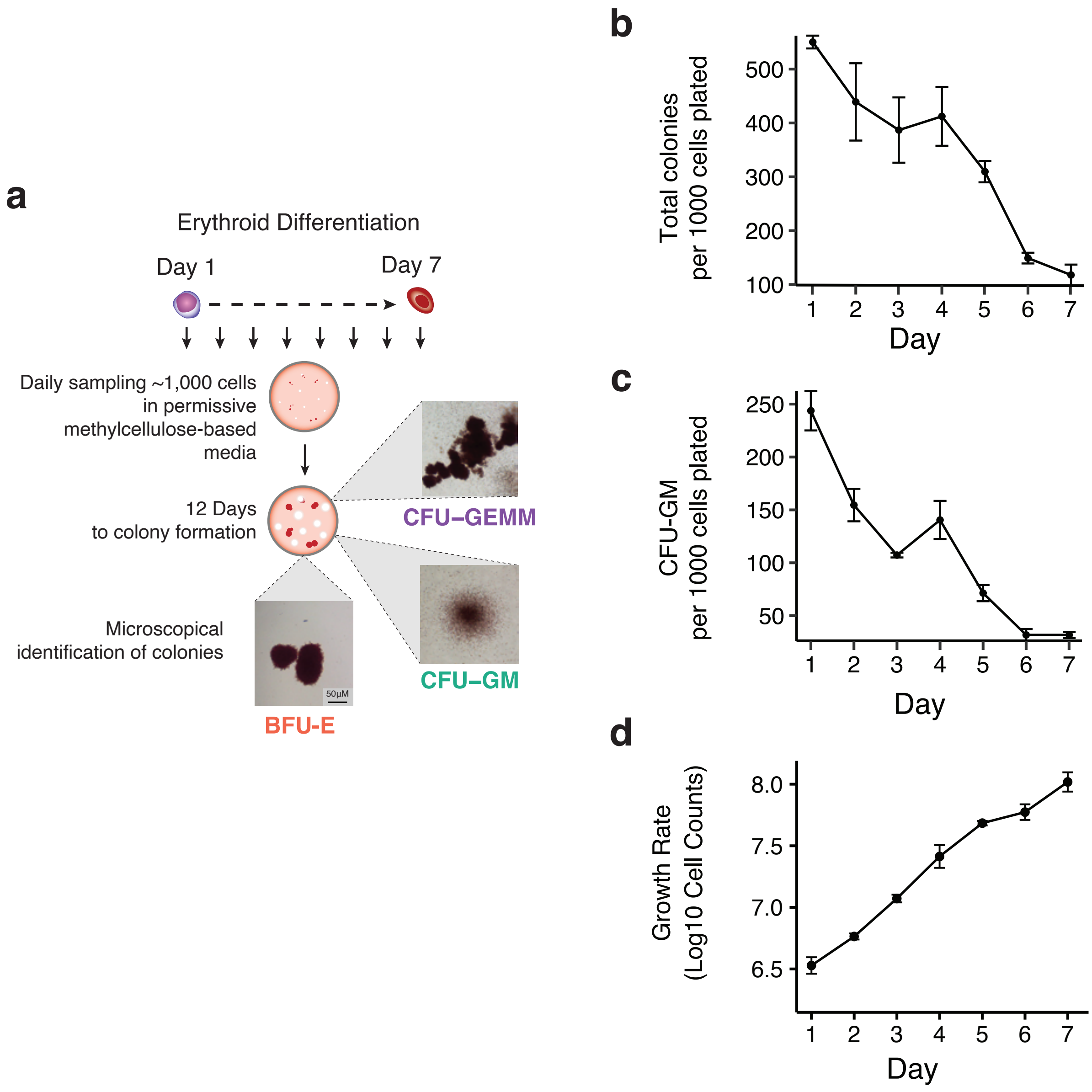

Supplementary Figure 12. Rapid changes in clonogenic potential observed during erythroid differentiation. (a) Schematic of the colony forming assay (MethoCult) during days 1-7 of ex vivo erythropoiesis along with representative microscope images of the detected types of colonies. (CFU-GEMM: Colony Forming Unit - Granulocyte/Erythroid/Macrophage/Megakaryocytic. CFU-GM: Colony Forming Unit - Granulocyte/Monocyte. BFU-E: Burst Forming Unit - Erythroid). (b) Changes in total clonogenic capacity during erythroid differentiation expressed as the number of colonies detected per 1,000 cells plated in methylcellulose assay. Data are represented as mean values of ±1 SEM n=4 experiments. (c) Granulocytic/monocytic (CFU-GM) progenitor frequency per 1,000 cells plated during erythroid differentiation. Data are represented as mean values of ±1 SEM n=4 experiments. (d) Cell expansion curve of the primary erythroid cultures from which cells were subjected to colony formation assays. The rate of growth is increasing monotonically over time. Values are average of n=3 experiments and errorbars denote ±1 SEM.

Supplementary Figure 13

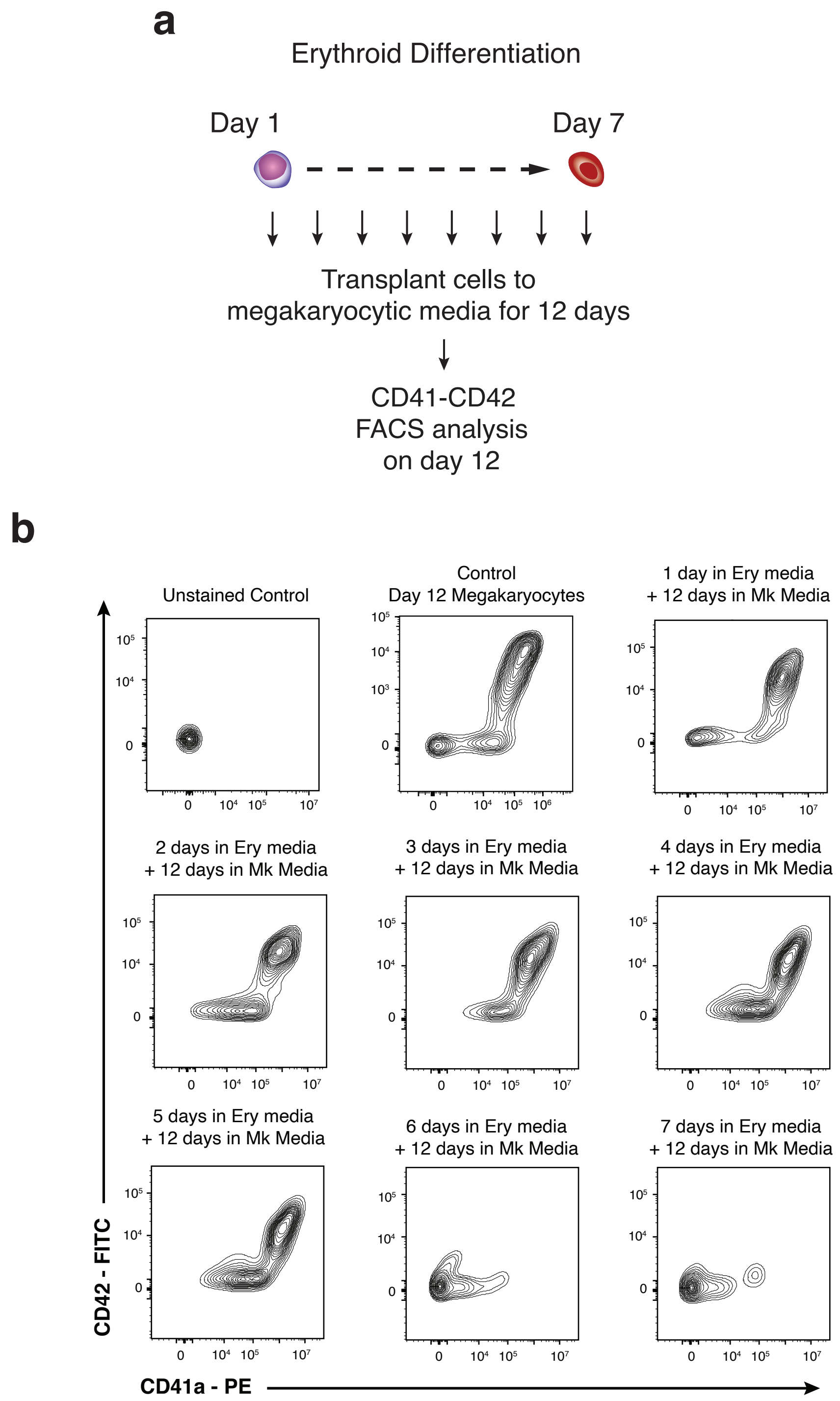

Supplementary Figure 13. A suspension-based assay to test for megakaryocytic potential during erythropoiesis. (a) Schematic of megakaryocytic lineage potential assay during days 1-7 of ex vivo erythropoiesis where cells sampled daily were transferred to secondary suspension megakaryocytic media for 12 days. (b) Expression scatterplots of CD41a (x-axis) and CD42b (y-axis) of the cultures initiated from day 1 to day 7 of erythropoiesis. FACS profile of CD41 expression of a control megakaryocytic culture on day 12 is included as control.

Supplementary Figure 14

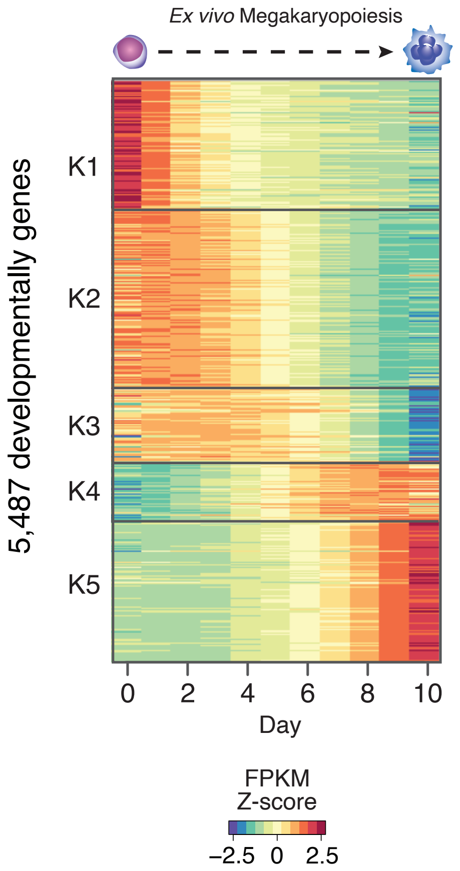

Supplementary Figure 14. *K*-means clustering of developmentally regulated genes during ex vivo megakaryocytic differentiation. Dense gene expression time-course during ex vivo megakaryopoiesis and linear regression analysis identifies 5,487 significantly changing transcripts organized in 5 clusters (K1-K5) by *K*-means clustering.

Supplementary Figure 15

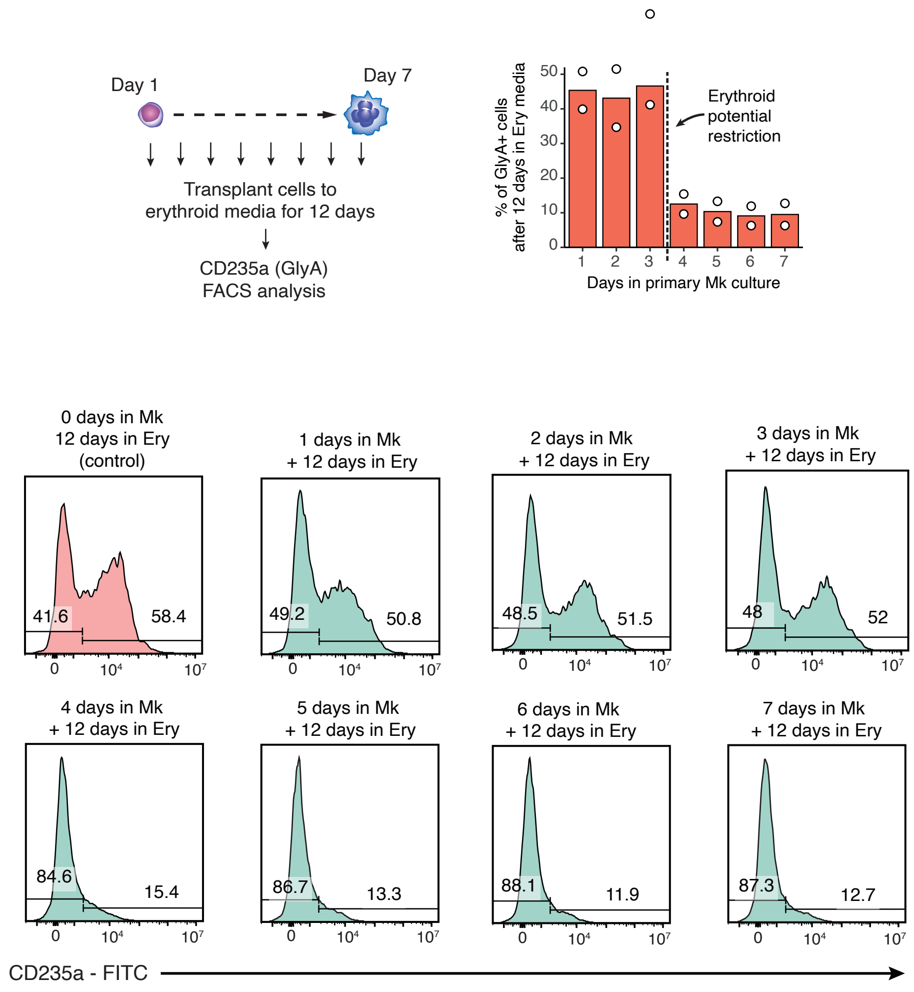

Supplementary Figure 15. Assessment of the erythroid potential along megakaryocytic differentiation in suspension assay. Frequency of mature erythroid cells (CD235a+) 12 days post transplantation into erythroid suspension culture of cells sampled from days 1-7 of primary megakaryocytic cultures.

Supplementary Figure 16

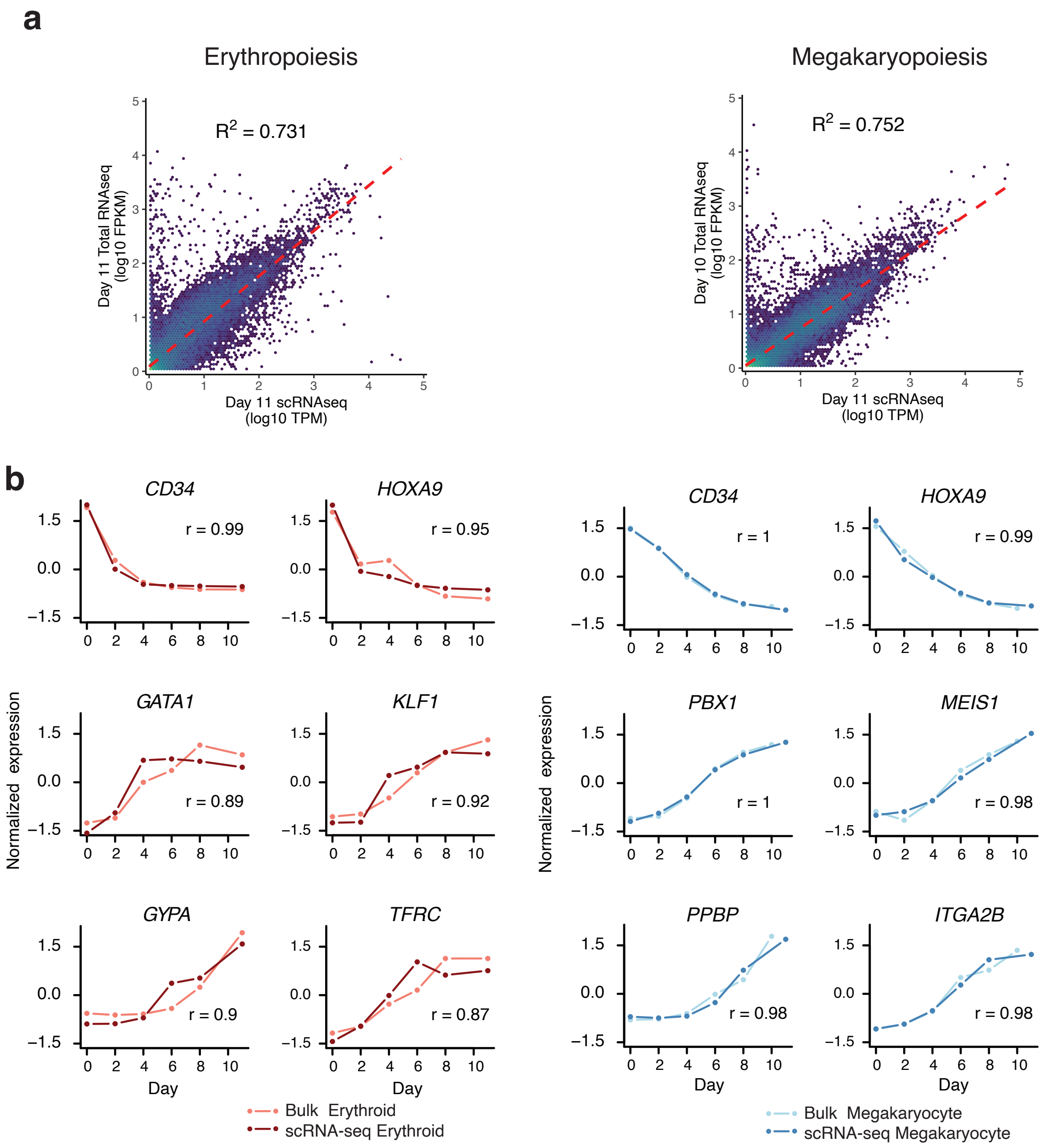

Supplementary Figure 16. Comparison of transcriptional dynamics along erythropoiesis and megakaryopoiesis between bulk and single-cell. (a) Scatterplots of scRNA-seq gene expression (TPM) against total RNA-seq (FPKM) values and the respective R2 values showing that data from the two experiments are highly concordant for erythrocytes (top) and megakaryocytes (bottom). Points are bins of individual genes where the color represents the number of points in a bin. (b) Representative examples of genes and their correlated expression between scRNA-seq and total RNA-seq experiments during erythropoiesis along with their Pearson correlation scores. (c) Representative examples of genes and their correlated expression between scRNA-seq and total RNA-seq experiments during megakaryopoiesis along with their Pearson correlation scores. (d) Hierarchical clustering of the samples using 10,000 highly variable, highly expressed genes displays data structure associated with sampling days and lineages.

Supplementary Figure 17

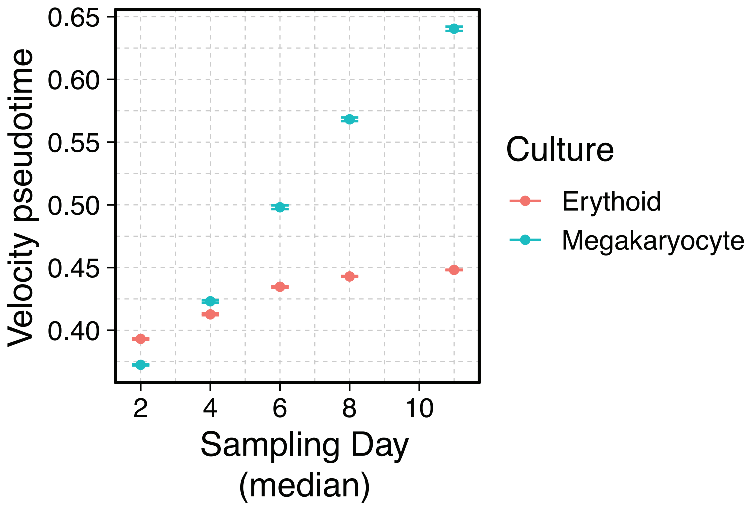

Supplementary Figure 17. Differential pseudotemporal dynamics between erythropoiesis and megakaryopoiesis. Mean ± standard error of velocity pseudotime of single cells sampled along erythropoiesis and megakaryopoeisis.

Supplementary Figure 18

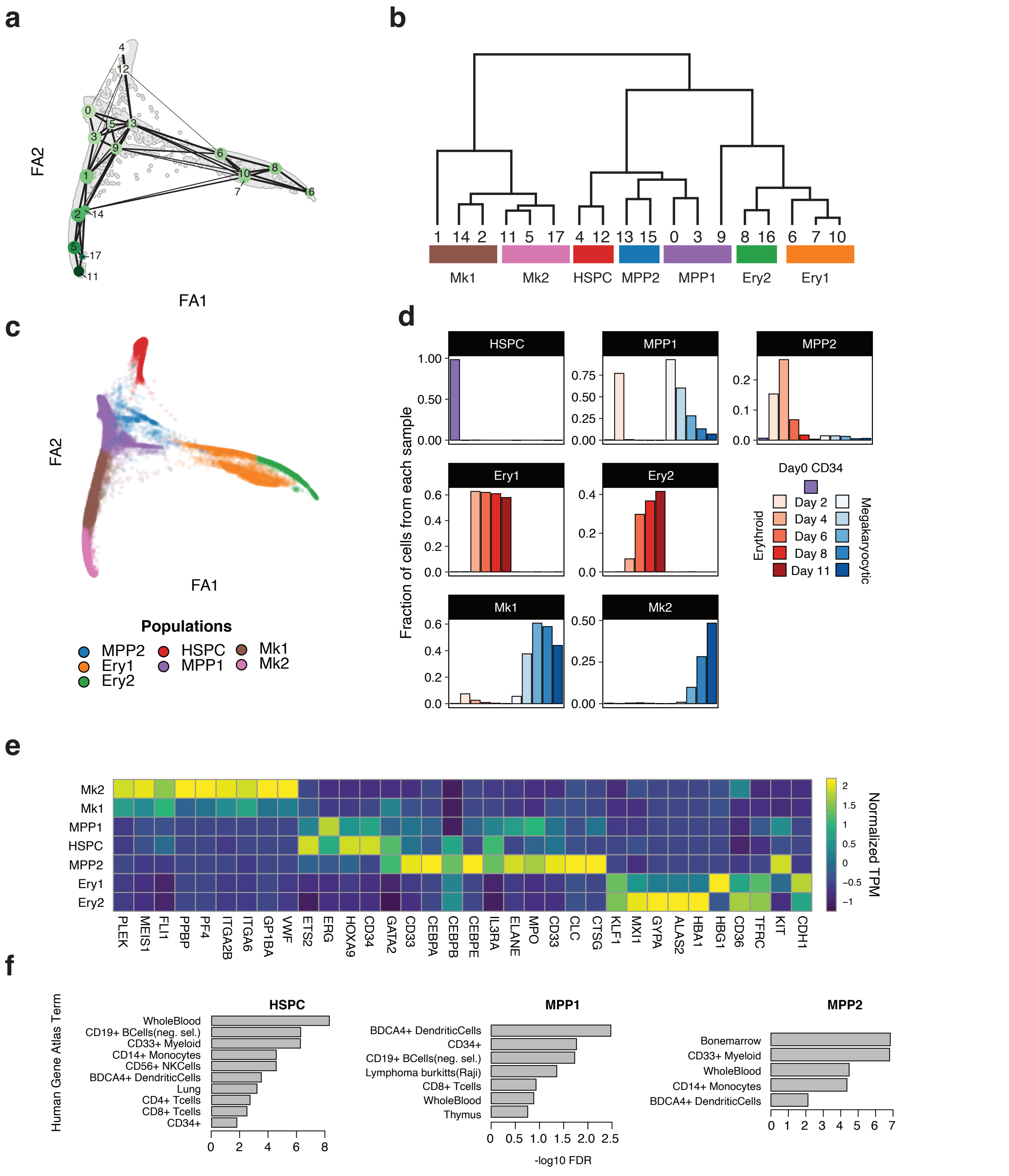

Supplementary Figure 18. Clusters of transcriptionally distinct cell states. (a) Representation of Leiden clusters (0-17) and their PAGA connectivities (black lines) on Force-Atlas projection. Clusters are colored by average pseudotime. Cluster size (number of cells per cluster) is denoted as the size of circles. (b) Dendrogram of the Euclidean distances between Leiden clusters collapsed into biologically relevant populations (colored bars). (c) Topology of identified populations on the Force-Atlas projection. (d) The composition of populations as fraction of total cells sampled from each timepoint and lineage. (e) Heatmap of the expression (Normalized TPM) of representative erythroid, HSPC, megakaryocytic, and myeloid across populations. (f) Top significantly enriched gene sets found in the marker genes for each of the early populations (HSPC, MPP1, MPP2).

Supplementary Figure 19

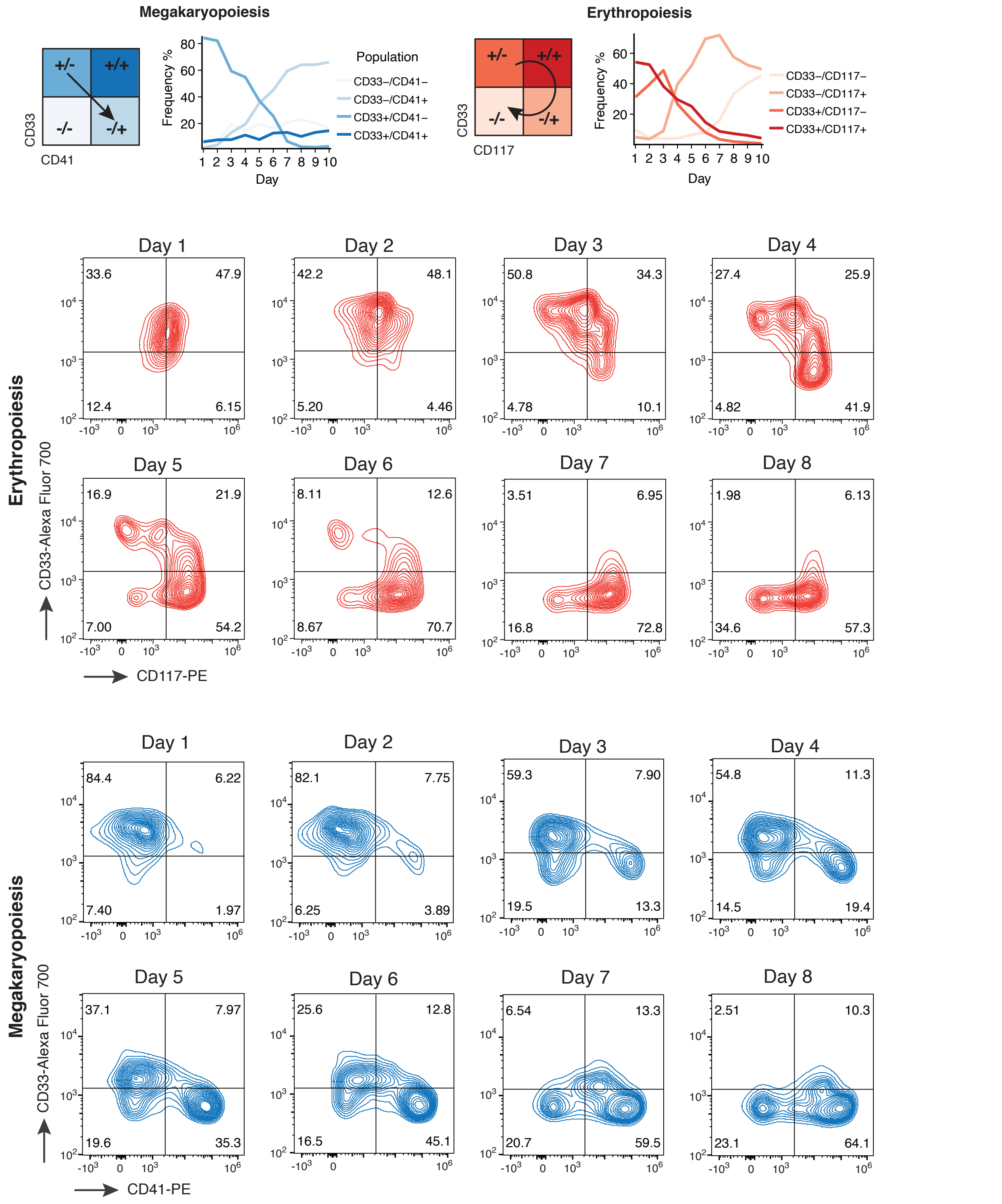

Supplementary Figure 19. Identification of myeloid populations during ex vivo erythropoiesis and megakaryopoiesis. Flow cytometry timecourse of the early myeloid marker CD33 (y-axis) during ex vivo erythroid (in red) and megakaryocytic (in blue) differentiation against CD117 (C-Kit) and CD41, respectively (x-axis).

Supplementary Figure 20

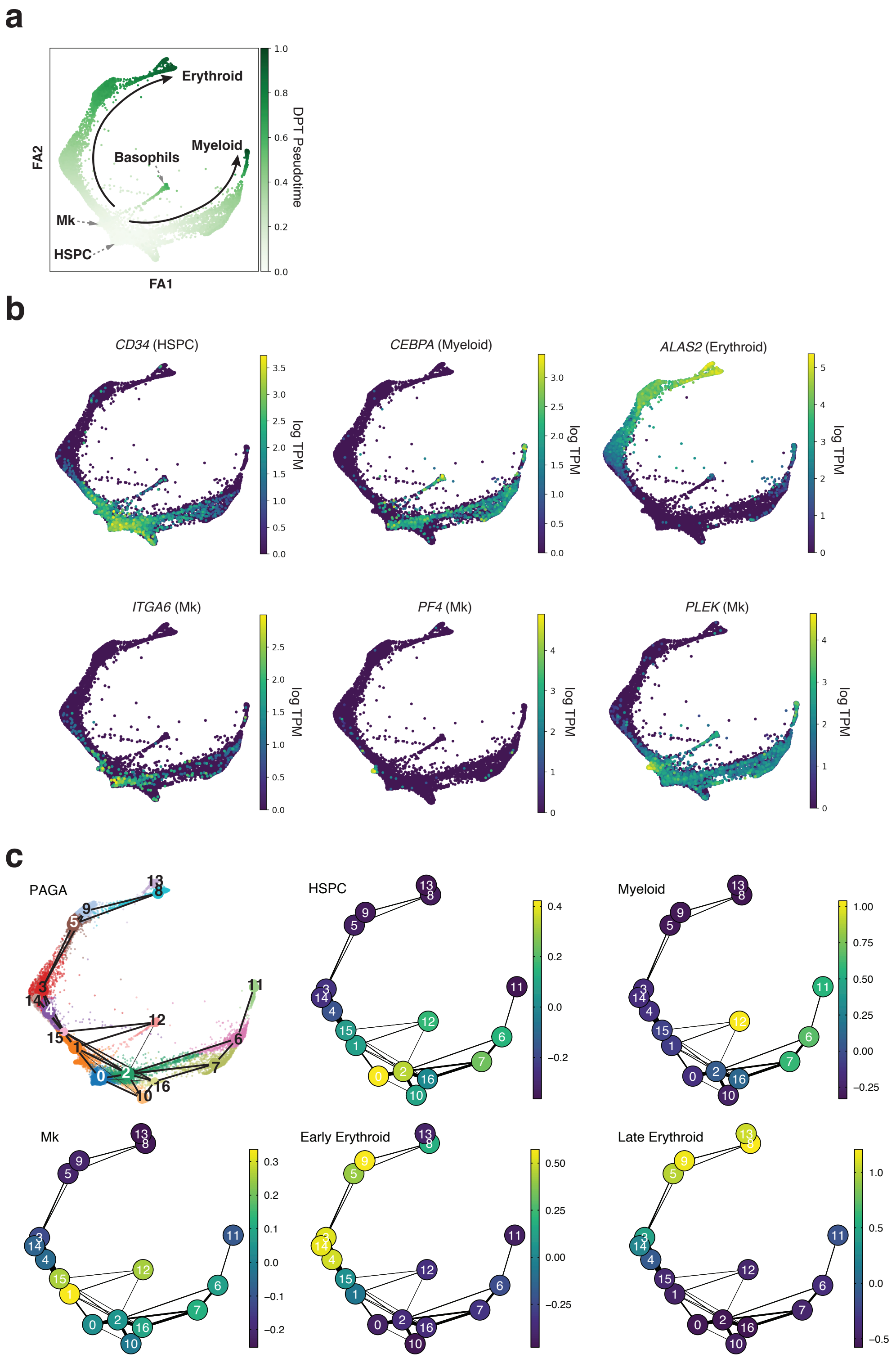

Supplementary Figure 20. Trajectory analysis and clustering of BM-derived hematopoietic progenitor populations. (a) Diffusion pseudotime annotation of BM-derived hematopoietic populations on Force-Atlas projection. Solid arrows annotate the erythroid and myeloid trajectories. (b) Gene expression patterns of representative HSPC, Myeloid, Erythroid and Mk genes. (c) Leiden clusters (0-16) with PAGA connectivities (black lines) and average expression (log TPM) profiles per cluster for lineage marker gene sets. *HSPC: HOXA9, PROM1, THY1, FOS, CD34*. Myeloid: *SPI1, MPO, GATA2, CD33, CEBPA, CEBPB, IL3RA, CTSG*. Early Erythroid: *TFRC, KIT, CD36, CDH1, KLF1*. Late Erythroid: *HBB, HBA2, MXI1, ENG, ALAS2, GYPA. Mk: PLEK, ELF1, FLI1, TBP, ITGA2B, PPBP, PF4, GP9, MEIS1.*

Supplementary Figure 21

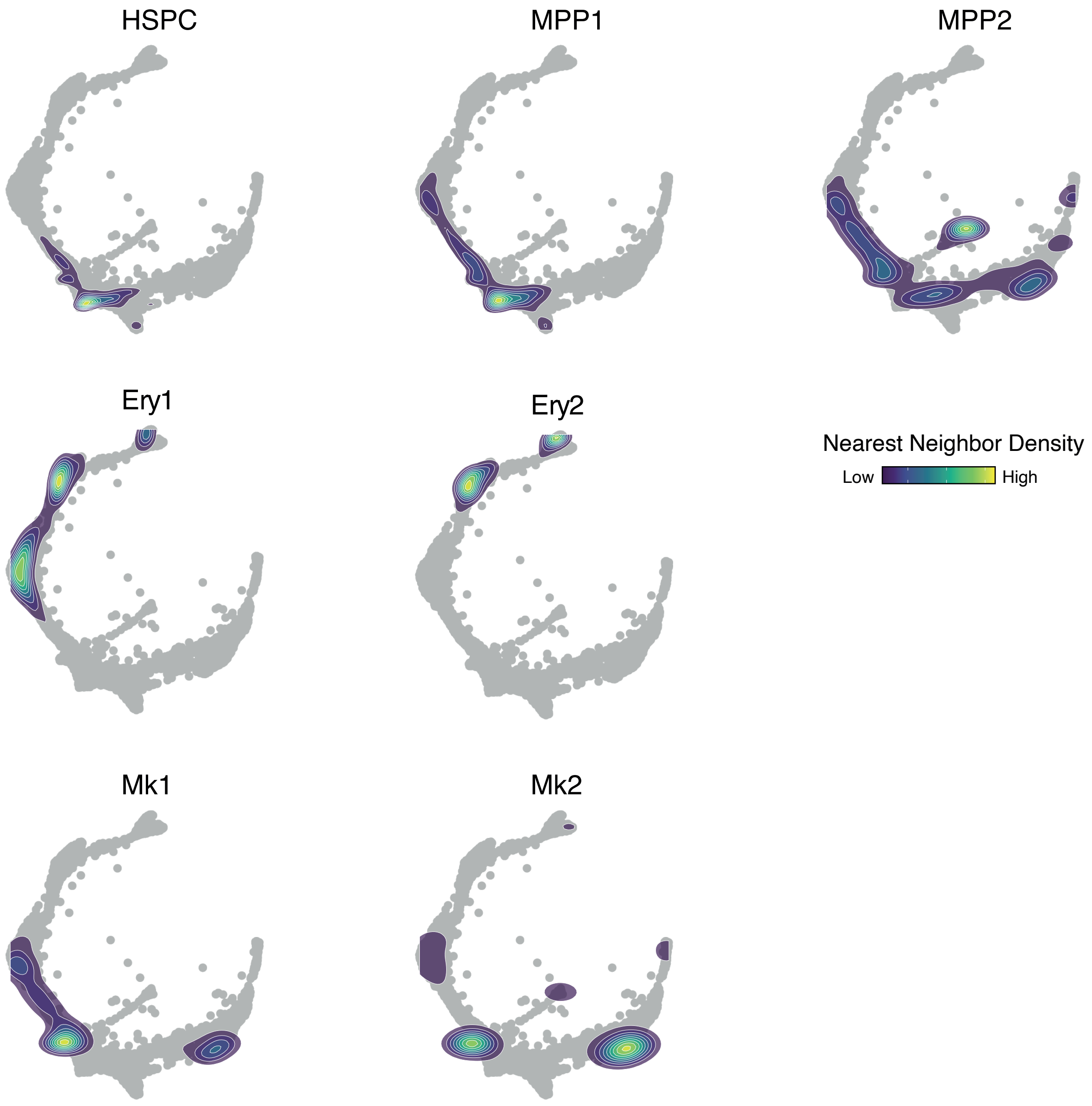

Supplementary Figure 21. Integrative analysis identifies corresponding states between ex vivo and bone marrow steady-state hematopoietic populations. Projection of *ex vivo* identified populations onto the FA map of bone marrow single cell RNA-seq data. 2D densities represent the *k*=20 nearest bone marrow neighbor cells for each of the *ex vivo* identified populations.

Supplementary Figure 22

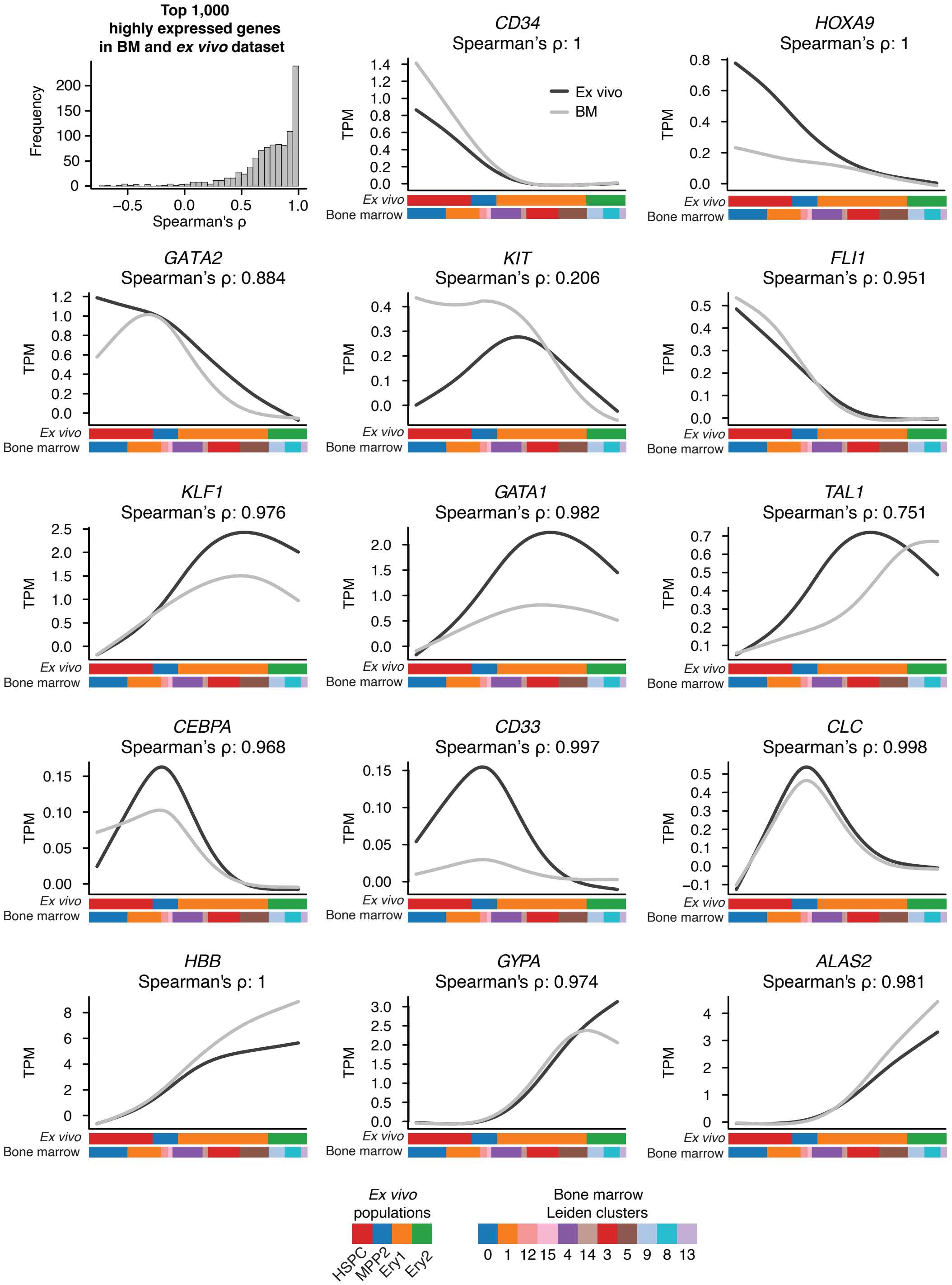

Supplementary Figure 22. Single-cell transcriptional dynamics of ex vivo erythropoiesis recapitulate erythroid population trajectories in the bone marrow. Histogram of correlation (Spearman's *ρ*) of scRNA-seq expression profiles of the top 1,000 highly expressed genes between ex vivo erythroid differentiation and bone marrow erythroid trajectories (top left panel). Gene profiles for a selection of marker genes from ex vivo erythroid trajectories and bone marrow erythroid population trajectories. Trajectories are inferred from PAGA transitions between clusters or populations whereby cells within are ordered by pseudotime.

TALEN Pair Name	TALEN Pair Sequence
HS2 left	TCAACGGGCATACACTAAGAAaatgaagtattttaatGACAGCAGGTAAAATCA
HS2 right	TTGATTTGCACTCCCGTctgccatgtttagtTAGGTTATTTCACCCAA
HS1 left	TGTTGGGGCTGGGTGATGAATacctgtggtttcattaTACTATATGTAGTGTGTGTTA
HS1 right	TAGCAATTCCAGTTTCTCTGgggattcctccccaGTGGGCTGGCGGTTGGA
Promoter left	TCTGATCCCAGGTCTTAGTGAgccaccggcggggcTGGGATTCGAACCCAGTGGAA
Promoter right	TTCGGGTCCTGAGGAGCGgagcggcctggaagccTCGCGCGCTCCGGACCCCCCA

Supplementary Table 1: TALEN pair sequences used to generate CDH1 knock-outs. Spacer sequence is annotated with lowercase

Primer Name	Sequence 5'-3'
CDH1 HS2 OUT F	ATGTCACATCAAGGATTCAACGG
CDH1 HS2 IN R	AGAGCAAGGGAATCAGGTGC
CDH1 HS2 OUT R	AGCCTTTGAGTCAGAGCCAC
CDH1 HS1 OUT F	TGTTGGGGCTGGGTGATGAAT
CDH1 HS1 OUT R	TTAATCCTTCAGGCAGTCTTGTCC
CDH1 PR OUT F	CAGCCAAGTGTAAAAGCCCT
CDH1 PR OUT R	TCCCCCTCGACTTGCACC

Supplementary Table 2: Primer sequences used to detect genetic deletions of target regions in CDH1 knock-outs.
