# Supplementary material for: Discrete regulatory modules instruct hematopoietic lineage commitment and differentiation
Source: Nat Commun. 2021 Nov 23;12:6790. doi: 10.1038/s41467-021-27159-x (PMC8611072; doi:10.1038/s41467-021-27159-x)
Supplement: Supplementary file 2 — Reporting Summary [file 41467_2021_27159_MOESM2_ESM.pdf]

nature portfolio

Corresponding author(s): Grigorios Georgolopoulos, Jeff Vierstra

Last updated by author(s): Oct 19, 2021

Reporting Summary

Nature Portfolio wishes to improve the reproducibility of the work that we publish. This form provides structure for consistency and transparency in reporting. For further information on Nature Portfolio policies, see our Editorial Policies and the Editorial Policy Checklist.

Statistics

For all statistical analyses, confirm that the following items are present in the figure legend, table legend, main text, or Methods section.

n/a	Confirmed	
☐	☒	The exact sample size (*n*) for each experimental group/condition, given as a discrete number and unit of measurement
☐	☒	A statement on whether measurements were taken from distinct samples or whether the same sample was measured repeatedly
☐	☒	The statistical test(s) used AND whether they are one- or two-sided<br>*Only common tests should be described solely by name; describe more complex techniques in the Methods section.*
☐	☒	A description of all covariates tested
☐	☒	A description of any assumptions or corrections, such as tests of normality and adjustment for multiple comparisons
☐	☒	A full description of the statistical parameters including central tendency (e.g. means) or other basic estimates (e.g. regression coefficient) AND variation (e.g. standard deviation) or associated estimates of uncertainty (e.g. confidence intervals)
☐	☒	For null hypothesis testing, the test statistic (e.g. *F*, *t*, *r*) with confidence intervals, effect sizes, degrees of freedom and *P* value noted<br>*Give P values as exact values whenever suitable.*
☒	☐	For Bayesian analysis, information on the choice of priors and Markov chain Monte Carlo settings
☒	☐	For hierarchical and complex designs, identification of the appropriate level for tests and full reporting of outcomes
☐	☒	Estimates of effect sizes (e.g. Cohen's *d*, Pearson's *r*), indicating how they were calculated

Our web collection on statistics for biologists contains articles on many of the points above.

Software and code

Policy information about availability of computer code

Data collection	Genomic sequencing data were directly downloaded from GEO or ENA. Data collection is detailed in the Methods section of the manuscript
Data analysis	DNase I sequencing alignment was performed using BWA sequence aligner v0.7.12<br>Genomic region manipulation was performed using BEDOPS v.2.4.35<br>Gene expression sequencing data was performed using STAR aligner<br>DNaseI hotspots were called using Hotspot2 (https://github.com/Altius/hotspot2).<br>Single-cell RNAseq analysis was performed using SCANPY (https://github.com/theislab/scanpy).<br>Single-cell RNA Velocity and trajectory analysis was performed using Velocyto (https://github.com/velocyto-team/velocyto.py) and scVelo (https://github.com/theislab/scvelo).<br>kNN analysis was performed using the FindNeighbors function from Seurat (https://github.com/satijalab/seurat).<br>HiC data preprocessing and normalization was performed using Cooler (https://github.com/open2c/cooler) and HiCExplorer (https://github.com/deeptools/HiCExplorer).<br>Anchor loops from HiC data were called using Mustache (https://github.com/ay-lab/mustache).<br>Python 2.7 and 3.5 as well as R version 3.4 and 3.6 were used to analyze the data.

For manuscripts utilizing custom algorithms or software that are central to the research but not yet described in published literature, software must be made available to editors and reviewers. We strongly encourage code deposition in a community repository (e.g. GitHub). See the Nature Portfolio guidelines for submitting code & software for further information.

Data

Policy information about availability of data

All manuscripts must include a data availability statement. This statement should provide the following information, where applicable:

- Accession codes, unique identifiers, or web links for publicly available datasets
- A description of any restrictions on data availability
- For clinical datasets or third party data, please ensure that the statement adheres to our policy

All sequencing data have been deposited to GEO under the series accession GSE182816 [https://www.ncbi.nlm.nih.gov/geo/query/acc.cgi?acc=GSE182816]. All processed data files available herein and relevant metadata are available on Zenodo [https://doi.org/10.5281/zenodo.5291737]. Adult erythroid Hi-C data was downloaded from GEO, series GSE102201 [https://www.ncbi.nlm.nih.gov/geo/query/acc.cgi?acc=GSE102201]. Adult CD43+ HSPC Hi-C data obtained from ENA, accession ERR436024 [https://www.ebi.ac.uk/ena/browser/view/ERR436024?show=reads]. Human bone marrow single-cell data was download from GEO using the following accessions: GSM3305359 [https://www.ncbi.nlm.nih.gov/geo/query/acc.cgi?acc=GSM3305359], GSM3305360 [https://www.ncbi.nlm.nih.gov/geo/query/acc.cgi?acc=GSM3305360], GSM3305361 [https://www.ncbi.nlm.nih.gov/geo/query/acc.cgi?acc=GSM3305361], GSM3305362 [https://www.ncbi.nlm.nih.gov/geo/query/acc.cgi?acc=GSM3305362], GSM3305363 [https://www.ncbi.nlm.nih.gov/geo/query/acc.cgi?acc=GSM3305363], GSM3305364 [https://www.ncbi.nlm.nih.gov/geo/query/acc.cgi?acc=GSM3305364], GSM3305365 [https://www.ncbi.nlm.nih.gov/geo/query/acc.cgi?acc=GSM3305365]

Field-specific reporting

Please select the one below that is the best fit for your research. If you are not sure, read the appropriate sections before making your selection.

[x] Life sciences [ ] Behavioural & social sciences [ ] Ecological, evolutionary & environmental sciences

For a reference copy of the document with all sections, see nature.com/documents/nr-reporting-summary-flat.pdf

Life sciences study design

All studies must disclose on these points even when the disclosure is negative.

Sample size	G-CSF mobilized, CD34-enriched (CD34+) peripheral blood mononuclear cells from three healthy donors (purchased from Fred Hutch Cancer Research Center, Seattle WA) to ensure statistical power for DNaseI-seq and RNA-seq experiments. For lineage potential assays results from 4 independent experiments were considered unless otherwise indicated. Experimental design and sample size for each experiment are detailed in the manuscript.
Data exclusions	No data were excluded in the present study.
Replication	All results were reliably reproduced in multiple independent experiments as indicated in respective sections within the Methods section or figure legends.
Randomization	Human CD34+ donors were randomly selected for sex, race and age.
Blinding	Blinding was not applicable to the study design of this work as it does not involve case/control specimens.

Reporting for specific materials, systems and methods

We require information from authors about some types of materials, experimental systems and methods used in many studies. Here, indicate whether each material, system or method listed is relevant to your study. If you are not sure if a list item applies to your research, read the appropriate section before selecting a response.

Materials & experimental systems

n/a	Involved in the study	
[ ]	[x]	Antibodies
[ ]	[x]	Eukaryotic cell lines
[x]	[ ]	Palaeontology and archaeology
[x]	[ ]	Animals and other organisms
[ ]	[x]	Human research participants
[x]	[ ]	Clinical data
[x]	[ ]	Dual use research of concern

Methods

n/a	Involved in the study	
[x]	[ ]	ChIP-seq
[ ]	[x]	Flow cytometry
[x]	[ ]	MRI-based neuroimaging

Antibodies

Antibodies used	All antibodies used for flow cytometry were diluted per manufacturer's instructions and all listed below as well as described in detail in the Methods section.

	PE anti-human CD117, 1:10 dilution. (Cat. No.: 340529, Clone 104D2, BD Biosciences)<br>FITC anti-human CD235a, 1:20 dilution (Cat. No.: 559943, Clone GA-R2/HIR2, BD Biosciences)<br>PE anti-human CD41, 1:10 dilution (Cat. No.: 555467, Clone HIP8, BD Biosciences)<br>APC anti-human CD42b, 1:10 dilution (Cat. No.: 551061, Clone HIP1, BD Biosciences)<br>Alexa Fluor 700 anti-human CD33, 1:20 dilution (Cat. No.: 561160, Clone WM53, BD Biosciences)<br>APC anti-human CD324 (E-Cadherin), 1:20 dilution (Cat. No.: 324108, Clone 67A4, Biolegend)
Validation	All antibodies are commercially available and are validated by the respective manufacturer.

Eukaryotic cell lines

Policy information about cell lines

Cell line source(s)	HUDEP-2 cells were kindly provided Ryo Kurita and Yukio Nakamura, Cell Engineering Division, RIKEN BioResource Center, Tsukuba, Ibaraki, Japan
Authentication	HUDEP-2 cell line was not authenticated
Mycoplasma contamination	All cell lines and primary cell cultures tested negative for mycoplasma
Commonly misidentified lines<br>(See ICLAC register)	None of the cell lines used in this manuscript are listed in the ICLAC Database of Cross-contaminated or Misidentified Cell Lines.

Human research participants

Policy information about studies involving human research participants

Population characteristics	Donor characteristics (age, sex, race) were not requested in the present study for randomization purposes. They are available upon request by the Cooperative Centers of Excellence in Hematology Core B (Fred Hutch Research Center, Seattle WA). According to the participation protocol (985.03), the age of all human peripheral blood mononuclear cell donors ranges between 18 and 70 years old.
Recruitment	Human subject recruitment and material collection was carried out by the Cooperative Centers of Excellence in Hematology Core B (Fred Hutch Research Center, Seattle WA). Recruitment process of human subjects is detailed in the participation protocol (985.03) available here: https://www.fredhutch.org/en/research/divisions/clinical-research-division/research/co-operative-center-for-excellence-in-hematology/hematopoietic-cell-procurement-and-resource-development/_jcr_content/root/responsivegrid/downloadpdf_1997229461/file.res/985.03%20Protocol.pdf
Ethics oversight	Cells were isolated under the protocol approved by the Fred Hutch Institutional Review Board (protocol no. 985.03), and in accordance with the Declaration of Helsinki. All donors have authorized the collection and informed consent was given to the biobank at the time of collection.

Note that full information on the approval of the study protocol must also be provided in the manuscript.

Flow Cytometry

Plots

Confirm that:

- [x] The axis labels state the marker and fluorochrome used (e.g. CD4-FITC).
- [x] The axis scales are clearly visible. Include numbers along axes only for bottom left plot of group (a 'group' is an analysis of identical markers).
- [x] All plots are contour plots with outliers or pseudocolor plots.
- [x] A numerical value for number of cells or percentage (with statistics) is provided.

Methodology

Sample preparation	Upon media removal, cell samples were incubated with staining solution where antibodies were diluted per manufacturer's recommendations. Details on sample preparation for flow cytometry are available in the Methods section.
Instrument	Samples were acquired with CytoFlex S (Beckman Coulter)
Software	Flow Cytometry sample acquisition was performed with CytExpert (Beckman Coulter). Analysis was performed with FlowJo (Becton Dickinson)
Cell population abundance	No sorting experiments were performed to require verification of cell population abundance

Gating strategy	All flow cytometry analyses were performed after gating on FSC-A/SSC-A > FSC-H/FSC-A (singlets)

☒ Tick this box to confirm that a figure exemplifying the gating strategy is provided in the Supplementary Information.
