# Supplementary material for: Discrete regulatory modules instruct hematopoietic lineage commitment and differentiation
Source: Nat Commun. 2021 Nov 23;12:6790. doi: 10.1038/s41467-021-27159-x (PMC8611072; doi:10.1038/s41467-021-27159-x)
Supplement: Supplementary file 3 — Description of Additional Supplementary Files [file 41467_2021_27159_MOESM3_ESM.pdf]

Description of Additional Supplementary Files

Supplementary Data 1. A tab-separated file listing the GRCh38 coordinates of all detected DHS (Hotspots FDR 5%) during *ex vivo* erythroid differentiation. mean is the average normalized DNase I density across 12 timepoints and 3 donors. l2fc is the log2 fold-change from the lowest daily density (3-donor average) to the highest observed. FDR is the false discovery rate of the likelihood ratio test between the explicit and the reduced linear regression model for DNase I density. Cluster is the *K*-means (k = 5) cluster assignment. DHS with NA assigned cluster were not considered as developmentally changing DHS (see methods).

Supplementary Data 2. A table of all the developmentally regulated transcripts during *ex vivo* erythroid differentiation. mean_FPKM is the average normalized FPKM between day 0 and 12 and across 3 donors. l2fc is the log2 fold-change from the lowest daily FPKM (3-donor average) to the highest observed. FDR is the false discovery rate of the likelihood ratio test between the explicit and the reduced linear regression model for gene expression. Cluster is the *K*-means (k = 5) cluster assignment.

Supplementary Data 3. A BED file with the TADs called at 10kb resolution from CD34 HSPC Hi-C data obtained from Misfud et al., 2015

Supplementary Data 4. A BED file with the TADs called at 10kb resolution from day 11 *ex vivo* derived erythroid progenitors form Hi-C data obtained from Huang et al., 2017.

Supplementary Data 5. Transcription factor motif enrichment information per DHS cluster. l2fc is the log2-fold change of the motif frequency in the test cluster against the rest. FDR is the corrected hypergeometric enrichment test p-value.

Supplementary Data 6. A table listing the links between DHS (GRCh38 coordinates) and genes, along with distance from TSS, Pearson correlation, correlation test p-value.

Supplementary Data 7. A bedgraph file with Mustache loops calculated from TADs at 10kb resolution from Day 11 *ex vivo* derived erythroid progenitors.

Supplementary Data 8. A table of all the developmentally regulated transcripts during *ex vivo* megakaryopoiesis. mean_FPKM is the average normalized FPKM between day 0 and 12 and across 3 donors. l2fc is the $\log_2$ fold-change from the lowest daily FPKM (3-donor average) to the highest observed. FDR is the false discovery rate of the likelihood ratio test between the explicit and the reduced linear regression model for gene expression. Cluster is the *K*-means (k = 5) cluster assignment.

Supplementary Data 9. Significant marker genes per single-cell population as identified by pairwise Wilcoxon-sum rank tests between populations. Test scores, p-values, adjusted p-values, log2 fold-change, and population contrasts are listed. Genes are filtered for adjusted p-value < 10-5 and absolute log2 fold-change > 1.
